# Genomic and Long-Term Transcriptomic Imprints Related to the Daptomycin Mechanism of Action Occurring in Daptomycin- and Methicillin-Resistant *Staphylococcus aureus* Under Daptomycin Exposure

**DOI:** 10.3389/fmicb.2020.01893

**Published:** 2020-08-14

**Authors:** Viviana Cafiso, Stefano Stracquadanio, Flavia Lo Verde, Irene De Guidi, Alessandra Zega, Giuseppe Pigola, Stefania Stefani

**Affiliations:** ^1^Department of Biomedical and Biotechnological Sciences, University of Catania, Catania, Italy; ^2^Department of Clinical and Experimental Medicine, University of Catania, Catania, Italy

**Keywords:** daptomycin-resistant methicillin-resistant *Staphylococcus aureus*, genomics, SNPome, virulome, resistome, RNA-seq, transcriptomics

## Abstract

Daptomycin (DAP) is one of the last-resort treatments for heterogeneous vancomycin-intermediate *Staphylococcus aureus* (hVISA) and vancomycin-intermediate *S. aureus* (VISA) infections. DAP resistance (DAP-R) is multifactorial and mainly related to cell-envelope modifications caused by single-nucleotide polymorphisms and/or modulation mechanisms of transcription emerging as result of a self-defense process in response to DAP exposure. Nevertheless, the role of these adaptations remains unclear. We aim to investigate the comparative genomics and late post-exponential growth-phase transcriptomics of two DAP-resistant/DAP-susceptible (DAP^R/S^) methicillin-resistant *S. aureus* (MRSA) clinical strain pairs to focalize the genomic and long-term transcriptomic fingerprinting and adaptations related to the DAP mechanism of action acquired *in vivo* under DAP pressure using Illumina whole-genome sequencing (WGS), RNA-seq, bioinformatics, and real-time qPCR validation. Comparative genomics revealed that membrane protein and transcriptional regulator coding genes emerged as shared functional coding-gene clusters harboring mutational events related to the DAP-R onset in a strain-dependent manner. Pairwise transcriptomic enrichment analysis highlighted common and strain pair-dependent Kyoto Encyclopedia of Genes and Genomes (KEGG) pathways, whereas DAP^R/S^ double-pair cross-filtering returned 53 differentially expressed genes (DEGs). A multifactorial long-term transcriptomic-network characterized DAP^R^ MRSA includes alterations in (i) peptidoglycan biosynthesis, cell division, and cell-membrane (CM) organization genes, as well as a *cid*B/*lyt*S autolysin genes; (ii) *ldh*2 involved in fermentative metabolism; (iii) CM-potential perturbation genes; and (iv) oxidative and heat/cold stress response-related genes. Moreover, a D-alanyl–D-alanine decrease in cell-wall muropeptide characterized DAP/glycopeptide cross-reduced susceptibility mechanisms in DAP^R^ MRSA. Our data provide a snapshot of DAP^R^ MRSA genomic and long-term transcriptome signatures related to the DAP mechanism of action (MOA) evidencing that a complex network of genomic changes and transcriptomic adaptations is required to acquire DAP-R.

## Introduction

Methicillin-resistant *Staphylococcus aureus* (MRSA) remains one of the major multidrug-resistant pathogens responsible for severe infections with high mortality rates (Stefani et al., 2015; Yoon et al., 2016; Britt et al., 2017). The subset of daptomycin-resistant (DAP^R^) *S. aureus* is of particular concern for the cumulative non-reversible metabolic changes demonstrated in resistant strains and for the difficulty in the treatment of severe infections (Bayer et al., 2013; Reed et al., 2019).

The DAP mechanism of action (DAP-MOA) has not been fully elucidated yet. The model of DAP-MOA proposes that DAP binds the cytoplasmic membrane leading to its permeabilization and depolarization caused by a loss of cytoplasm potassium ions (Allen et al., 1987). This mechanism, which can account for DAP’s bactericidal effect, would correlate with several changes in the membrane components, for example, with the level of phosphatidylglycerol. The complex mechanism of DAP resistance (DAP-R) was reviewed by Miller et al. (2016). Briefly, *S. aureus* can adopt different strategies to resist DAP. The first is to alter the net cell-surface charge, preventing the positively charged DAP-calcium binding to the cell envelope by electrostatic repulsion, mainly due to *dlt* over-expression—increasing the alanylation rate of the wall teichoic acids (Yang et al., 2009; Cafiso et al., 2014)—and to *mpr*F mutations increasing the amount of the positively charged lysyl-phosphatidylglycerol on the outer membrane, conferring a “gain-of-function” and positively increasing the cell-envelope charge (Rubio et al., 2011; Bayer et al., 2015; Ernst et al., 2018; Sabat et al., 2018; Ernst and Peschel, 2019). The second is the alteration of membrane phospholipid composition, with decreased phosphatidylglycerol amount, or changes in membrane fluidity interfering with DAP binding and oligomerization (Jones et al., 2008; Kilelee et al., 2010; Mishra et al., 2011; Zhang et al., 2014). A third mechanism is the involvement of global regulatory genes modulating cell-envelope stress and maintenance, affecting the expression of the cell-wall (CW) “stimulon” (Utaida et al., 2003; Cafiso et al., 2012). Specifically, VraSR operon (Muthaiyan et al., 2008; Mehta et al., 2012; Sabat et al., 2018; Taglialegna et al., 2019), orthologous to the *Bacillus subtilis* LiaSR (Jordan et al., 2006), was involved and up-regulated by vancomycin and DAP exposure, and associated with CW biosynthesis via transcription of PBP2 (penicillin-binding protein 2), *tag*A (wall teichoic acids-synthesis), *prs*A (a-chaperone), and *mur*Z (UDP-*N*-acetylglucosamine-enolpyruvyl transferase) (Kuroda et al., 2003; Mwangi et al., 2007). YycFG (also named WalKR), among the two-component regulatory systems (TCRSs), was implicated in the control of the peptidoglycan biosynthesis through the regulation of LytM and AtlA (Dubrac and Msadek, 2004; Fukushima et al., 2011). A YycFG down-regulation was associated with a decreased peptidoglycan turnover, augmented cross-linking, increased glycan chain length, and resistance to lysis by Triton X-100 (Dubrac et al., 2007). YycG senses changes in membrane fluidity and responds by adjusting CW cross-linking, compensating the stresses caused by osmotic pressure (Türck and Bierbaum, 2012). In DAP^R^ strains, *yycFG* can accumulate mutations (Friedman et al., 2006; Howden et al., 2011), impairing the YycFG function and altering CW homeostasis to survive the DAP action, that is, lowering peptidoglycan turnover and increasing cross-linking. Mutations in the RNA polymerase *rpo*B and *rpo*C subunits were associated with DAP-R (Friedman et al., 2006). A621E mutation in RpoB was associated with an increased expression of the *dlt*-operon and correlated with an increase in positive cell-surface charge, whereas A621E and A477D substitutions were linked to an increased CW biosynthesis and thickness (Cui et al., 2010; Bæk et al., 2015).

Different cellular metabolic shifts, including a decreased activity of the tricarboxylic acid cycle (TCA), were described in DAP^R^ strains (Fischer et al., 2011; Gaupp et al., 2015).

This investigation aimed to face the analysis of DAP-R mechanisms in MRSA strains on the basis of the acquired knowledge and considering new aspects. First, the “already known” DAP-R mechanisms (*dlt* over-expression, *mprF* mutations, and increased net positive cell-surface charge) do not always correlate with changes in DAP minimum inhibitory concentrations (MICs) (Mishra et al., 2014); second, during an infection, the bacteria elapse long periods in limited/arrested growth as in the late growth phases (Kolter et al., 1993); third, DAP is also active on non-dividing and not metabolically active bacteria (Mascio et al., 2007; McCall et al., 2019).

On this rationale, our study investigated genomics and, for the first time, the comparative late growth-phase transcriptomics of clinical DAP^R^ versus DAP^S^ isogenic MRSA by using whole-genome sequencing (WGS), RNA-seq, and bioinformatics to reveal their genomic traits as well as the long-term fingerprinting and adaptations related to DAP-MOA occurring in the acquisition of DAP-R by DAP^S^ isolates.

These new findings could advance the knowledge in the field, revealing the genomic and long-term transcriptomic key features acquired *in vivo* by the DAP^R^ versus their parental isogenic DAP^S^ ancestor under DAP exposure and maintained after DAP removal, becoming stable traits characterizing DAP^R^ MRSA.

Our data showed that DAP-MOA-related strain-dependent non-synonymous single-nucleotide polymorphisms (nsSNPs) in cell membrane (CM), structural/functional protein, and transcriptional regulator coding genes, combined with a complex transcriptional network, appeared as key factors characterizing DAP^R^ MRSA versus DAP^S^ parents.

## Materials and Methods

### Bacterial Strains

Two *S. aureus* epidemiologically unrelated isogenic strain pairs of DAP^S^ (1A, 3A) and DAP^R^ MRSA (1C, 3B) isolated under DAP therapy, previously collected from patients hospitalized in two Italian hospitals, were investigated. The two isogenic strain pairs were provided by two different Italian hospitals (Cafiso et al., 2014); consequently, an ethical approval was not necessary as per institutional and national guidelines and regulations. Specifically, the 1A and 1C strains were isolated in Palermo by a skin infection, whereas the 3A and 3B strains were isolated by a soft tissue infection in a patient in Bergamo. Each pair included an initial pre-DAP therapy strain (1A and 3A) and its isogenic isolate after development of DAP-R during DAP administration. Each strain pair was previously assigned to the same pulsotype, multilocus sequence typing (MLST) type, SCCmec type, and *agr* group (Cafiso et al., 2014). The D-alanylated wall teichoic acid content, the lysinylated-phosphatidylglyderol amount, the CM fluidity, and the susceptibility to prototypic cationic host defense peptides of platelet and leukocyte origins were previously determined (Mishra et al., 2014). Finally, exponential or stationary growth-phase expression and SNPs in the “already DAP-R associated genes,” *mpr*F and *dlt*A, were previously reported (Table 1) (Cafiso et al., 2014; Mishra et al., 2014).

**TABLE 1:** Phenotypic and molecular characterization of the MRSA strain pairs.

Strains	DAP phenotype	MICs (mg/L)				Glycopeptide heteroresistance	Molecular characterization						DAP-R versus DAP-S expression		Virulome	Resistome	Resistance gene SNPs			
								
		OXA	DAP	VAN	TEC		PFGE	MLST	SCC *mec*	spa-type	agr-type	Plasmids	*dltA*	*mprF*			Gene	Nucleotide change	AA change	Resistance
1A	DAP^S^	32	<0.25	1	<0.25	VSSA	A 1/*Apa*I	398	IVa	t1939	I	pDLK1 (*erm*C)	–	–	*spa*, *aur*, *cap8*, *adsA*, *cna*, *clpB*, *coa*, *ebp esxA*, *fnbA*, *geh*, *hys*A, *lip*, *map*, *sbi*, *srt*B, vWbp *clfA/B esaA/B essA/B/C hla*, *hlb*, *hld hlg*A, *hlg*B, *hlg*C *ica*RABCD *isd*A-G *sdr*C/D/E *ssp*A/B/C	Aminoglycoside-*R* (*aac*6′-*aph*2″) β-Lactam-*R* (*mec*A) Fosfomycin-*R* (*fos*D) Macrolide-*R* (*erm*C) Macrolide-*R* (*vga*A)V Phenicol-*R* (*fex*B) Tetracycline-*R* (*tet*38) Tetracycline-*R* (*tet*M) Trimethoprim-*R* (*dfr*C)	***rpo*B *grl*A**	GCT → GAT TCC → TTC	A477D S80F	**Rifampicin Ciprofloxacin**
1C	DAP^R^	64	4	2	2	hGISA	A 1/*Apa*I	398	IVa	t1939	I	pS194 (*str*) pDLK1 (*erm*C)	↑	↑**^1^**	*spa*, *aur*, *cap8*, *adsA*, *cna*, *coa*, *ebp esxA*, *clpB*, *geh*, *hys*A, *lip*, *map*, *sbi*, *srt*B, vWbp *clfA/B esaA/B essA/B/C fnbA/B hla*, *hlb*, *hld hlg*A, *hlg*B, *hlg*C *ica*RABCD *isd*A-G, *sdr*C/D/E *ssp*A/B/C	Aminoglycoside-*R* (*aac*6′-*aph*2″) Aminoglycoside-*R* (*str*) β-Lactam-*R* (*mec*A) Fosfomycin-*R* (*fos*D) Macrolide-*R* (*erm*C) Macrolide-*R* (*vga*A)V Phenicol-*R* (*fex*B) Tetracycline-*R* (*tet*38) Tetracycline-*R* (*tet*M) Trimethoprim-*R* (*dfr*C)	***rpo*B *grl*A**	GCT → GAT TCC → TTC	A477D S80F	**Rifampicin Ciprofloxacin**
3A	DAP^S^	2	0.5	1	16	hGISA	G1/*Sma*I	8	IVc	t008	I	SAP063A (*bla*Z)	–	–	*spa*, *aur*, *cap8*, *adsA*, *cna*, *coa*, *ebp esxA*, *clpB*, *geh*, *hys*A, *lip*, *map*, *sbi*, *srt*B, vWbp *clfA/B esaA/B essA/B/C fnbA/B hla*, *hlb*, *hld hlg*A, *hlg*B, *hlg*C luKF-PV, *luk*D, *luk*E *ica*RABCD *isd*A-G *sdr*C/D/E *ssp*A/B/C *spl*A, *spl*B, *spl*E *sed*, *sej*, *ser*	β-Lactam-*R* (*bla*Z) β-Lactam-*R* (*mec*A) Tetracycline-*R* (*tet*38) Fosfomycin-*R* (*fos*D)	***grlAgrlBgyrA***	TCC → TAC CCT → TCT TCA → TTA	S80Y P585S S84L	**Ciprofloxacin Ciprofloxacin Ciprofloxacin**
3B	DAP^R^	16	4	2	2	qVISA	G2/*Sma*I	8	IVc	t008	I	SAP063A (*bla*Z) pDLK1 (*erm*C)	↑	↓**^2^**	*spa*, *aur*, *cap8*, *adsA*, *cna*, *coa*, *ebp esxA*, *clpB*, *geh*, *hys*A, *lip*, *map*, *sbi*, *srt*B, vWbp *clfA/B esaA/B essA/B/C fnbA/B hla*, *hlb*, *hld hlg*A, *hlg*B, *hlg*C luKF-PV, *luk*D, *luk*E *ica*RABCD *isd*A-G *sdr*C/D/E *ssp*A/B/C *spl*A, *spl*B, *spl*E *sed*, *sej*, *ser*	β-Lactam-*R* (*bla*Z) β-Lactam-*R* (*mec*A) Tetracycline-*R* (*tet*38) Fosfomycin-*R* (*fos*D) Aminoglycoside-*R* (*aac*6′-*aph*2″) Macrolide-*R* (*erm*C)	***rpoBgrlAgrlBgyrA***	CAT → TAT TCC → TAC CCT → TCT TCA → TTA	S84L S80Y P585S S84L	**Rifampicin Ciprofloxacin Ciprofloxacin Ciprofloxacin**

*^1^S295L AA changes in MprF; ^2^no mutation in mprF; ↑, over-expression; ↓, under-expression; MRSA, methicillin-resistant Staphylococcus aureus; DAP, daptomycin; MICs, minimum inhibitory concentrations; DAP-R, daptomycin resistant; DAP-S, daptomycin susceptible; SNP, single-nucleotide polymorphism; PFGE, pulsed-field gel electrophoresis; MLST, multilocus sequence typing.*

### Whole-Genome Sequencing

Whole-genome sequencing was performed using the Illumina MiSeq sequencing system.

### DNA Extraction and Illumina Shotgun Paired-End Library Preparation

Genomic DNA was extracted using PureLink Genomic DNA Mini Kit (Invitrogen) according to the manufacturer’s protocol. The quality of the DNA was assessed using Qubit and its concentration ascertained by Picogreen (Life Technologies). Paired-end (PE) reads libraries were prepared by Nextera XT DNA Library Prep Kit (Illumina, San Diego, CA, United States) following the manufacturer’s protocol, and their quality evaluation was performed as previously published (Cafiso et al., 2019). The indexed libraries were quantified as previously published (Cafiso et al., 2019) pooled at a final concentration of 2 nM and used for Illumina MiSeq sequencing with a PE 300 (2 × 300 bp). Raw reads were processed using FastQC (v.0.11.7) to assess data quality. Cutadapter tool (v.1.16), implemented in Python (v.3.5.2), was used to remove residual PCR primer and to filter low-quality bases (*Q*_score < 30) and short reads (<150 bp). The filtered trimmed reads were included in the downstream analysis. The total number of PE reads was reported with the estimated coverage in Supplementary Figure S1.

### *De novo* Genome Assembly

*De novo* genome assembly was performed using SPAdes software (v3.12.0), producing a contig file for each sample. Post-assembly controls and metrics were generated using Quast (v.4.6.3). Supplementary Figure S2 shows *de novo* Genome Assembly Report.

### Genome Alignments

Genome alignments were per performed by Mauve (Multiple Genome Alignments) (v.2.4.0).

### Gene Annotation

The assembled scaffolds were processed using Prokka (v.1.12) software.

### DNA-Sequencing Data Accession Number

The genomic reads were deposited in the National Center for Biotechnology Information (NCBI) Genome database in the Sequence Read Archive (SRA) under study accession no. SRP166981 (BioProject: PRJNA498510).

### Single-Nucleotide Polymorphisms

Single-nucleotide polymorphisms calls were carried out from the PE library raw reads as previously published (Cafiso et al., 2019). Briefly, Illumina raw reads were trimmed using Trimmomatic (v.0.38), requiring a minimum base quality of 20 (Phred scale) and a minimum read length of 36 nucleotides, and aligned by “Bwa mem” (v.0.7.17). For detection of SNPs, and insertions and deletions (indels), each.bam file was sorted using Samtools (v.1.9), and duplicated reads were marked using the Picard Mark Duplicates utility. Complex variants, SNPs, and indels were detected using “Freebayes” (v.1.2.0), which required a minimum mapping quality of 30 (Phred scale) and a minimum base quality of 20. CSI phylogeny, a Center for Genomic Epidemiology service^1^, was used to identify the closest relationships between the strain pairs and four different reference genomes (RefGen) deposited in GenBank, that is, *S. aureus* NCTC8325 (CP000253.1), *S. aureus* USA 300 (CP000255.1), *S. aureus* MU50 (BA000017.4), and *S. aureus* ST398 (NC_017333.1).

*Staphylococcus aureus* NCTC8325 (CP000253.1) was selected as common RefGen for SNP mapping in agreement to the data obtained. The graphical output was a Phylogenetic tree shown in Supplementary Figure S3.

The majority of the sequenced reads was properly aligned with the corresponding reference genome, in detail, 90.98% for 1A, 91.08% for 1C, 96.45% for 3A, and 95.81% for 3B.

To select SNPs present in the DAP^R^ strains, all SNPs were computationally filtered in both pairwise mode and double-cross filtering to find out if only nsSNPs present simultaneously in both two DAP^R^ strains and the DAP^S^ parents.

### Genomic Epidemiology

Whole-genome sequencing raw data were analyzed to investigate the genomic epidemiology with ResFinder (v.3.2) and Point Finder (v.3.1.0) services for the detection of the acquired antimicrobial resistance (AMR) genes and to detect the known nsSNPs related to AMR using a 98% threshold for nucleotide sequence identity and 60% for the minimum length coverage (Zankari et al., 2012). MLST was genomically checked using the MLST (v2.0) (Larsen et al., 2012), spa-typing was confirmed by spaTyper (v.1.0) (Bartels et al., 2014), the identification of acquired virulence genes was performed by VirulenceFinder (v.2.0) (Joensen et al., 2014), and the plasmid strain profile was investigated by PlasmidFinder server (v.2.1) (Carattoli et al., 2014). All tools are available on the Center for Genomic Epidemiology website^1^. The genomic epidemiology of strain pairs was also analyzed by Nullarbor pipeline^2^.

### Core Genome Single-Nucleotide Polymorphisms

The core genome SNP (cgSNP) detection shared among all strains was computationally carried out by Nullarbor^2^.

### Enrichment Analysis

Enrichment analysis was performed by DAVID (v.6.8) using the default high stringency parameter set^3^ (Huang et al., 2009a, b).

### Genomic Single-Nucleotide Polymorphism Effect Prediction

The prediction of the whole-genome SNPs (SNPome) effects was performed by SnpEff (v.4.3T), a genomic variant annotation and functional effect prediction toolbox. High (HI), low (LI), moderate (MI), and modifier impacting (MFI) effects were assigned according to the criteria previously published and in use in the tool^4^ (Cingolani et al., 2012). High impact: the variant is assumed to have disruptive impact in the protein, probably causing protein truncation and loss of function or triggering nonsense mediated decay. Low impact: the variant is assumed to be mostly harmless or unlikely to change protein behavior. Moderate impact: the variant is a non-disruptive variant that might change protein effectiveness. Modifier impact: the variant is a usually non-coding variant or variants affecting non-coding genes where predictions are difficult or there is no evidence of impact.

### RNA-Seq

#### RNA-Seq Bacterial Cultures

For the construction of all RNA-seq libraries, an aliquot of an overnight culture was diluted 1:50 in 30 ml of brain heart infusion (BHI) in a sterile 50-ml flask (OD_600 nm_ 0.05) to obtain an approximately 5 × 10^5^ CFU/ml inoculum for each strain. Cells were grown under shaking at 250 rpm with normal atmospheric conditions at 37°C and harvested in the late post-exponential growth phase (1.5–4 × 10^10^ CFU/ml, ∼18 h). RNA extraction started immediately after cell harvesting to maintain RNA integrity. The cell density was determined by colony counting after plating onto Mueller–Hinton (MH) agar and incubation. Growth curves of the four DAP-free MRSA strains were shown in the Supplementary Figure S4.

#### RNA-Seq Libraries

RNA-seq was performed using the Illumina MiSeq sequencing system, and biological replicates were performed using two different libraries, a single-end library with 50-bp reads [Short-Insert Library (SI)] and a PE read library with 150-bp reads [Tru-Seq Library (TS)] starting from two different RNA extractions according to the following protocols as a strategy to optimize the collected RNA-seq data (Pereira et al., 2017; Cafiso et al., 2019).

#### Tru-Seq Library RNA Extraction

RNA was extracted using the NucleoSpin RNA (Macherey-Nagel, Dueren, Germany) kit following the manufacturer’s protocol with minor modifications. Pellets were lysed by the bead-beating procedure with 50 μl of RA1 buffer. Then 3.5 μl of β-mercaptoethanol was added, and the lysates were filtered through NucleoSpin Filters. The RNA-binding condition was adjusted by adding 70% ET-OH to the lysates, and the RNA was extracted with TRIzol reagent (Invitrogen) according to the manufacturer’s protocol. The total RNA quality was verified using a 2200 TapeStation RNA Screen Tape device (Agilent, Santa Clara, CA, United States), and its concentration was ascertained using an ND-1000 spectrophotometer (NanoDrop, Wilmington, DE, United States). The Agilent TapeStation 2200 system, an automated instrument for nucleic acid gel electrophoresis, assigned RNA integrity number (RIN) values ranging from 1 to 10, with 10 being the highest quality. Only samples with preserved 16S and 23S peaks and RIN values > 8 were used for the library’s construction. The RIN values > 8 indicate intact and high-quality RNA samples for downstream applications as previously published (Fleige and Pfaffl, 2006). Ribosomal RNA was removed using the Bacteria Ribo-Zero rRNA Removal Kit from 2 μg of RNA. The depleted RNA was used for the Illumina Truseq RNA stranded kit without PolyA enrichment. The obtained libraries were evaluated with high-sensitivity D1000 screen Tape (Agilent Tape Station 2200), and the indexed libraries quantified with the ABI9700 qPCR instrument using the KAPA Library Quantification Kit in triplicates was according to the manufacturer’s protocol (Kapa Biosystems, Woburn, MA, United States). From the pooled library, 2-nM final concentrations were used for sequencing with a 150 PE read sequencing module (Cafiso et al., 2019).

#### Short-Insert Library RNA Extraction

RNA was extracted with TRIzol reagent (Invitrogen) according to the manufacturer’s protocol. After ribosomal depletion, sequencing libraries were prepared using the Illumina mRNA-seq sample preparation kit following the supplier’s instructions except that total RNA was not fragmented and double-stranded cDNA was size-selected (100–400 bp) to maximize the recovery of small size RNA.

The prepared libraries were evaluated with high-sensitivity D1000 screen Tape (Agilent Tape Station 2200) as described for the TS Library. The indexed libraries were quantified in triplicate with the ABI7900 qPCR instrument using the KAPA Library Quantification Kit, according to the manufacturer’s protocol (Kapa Biosystems, Woburn, MA, United States). From the pooled library, 5 μl at a final concentration of 4 nM was used for MiSeq sequencing with an A single-end stranded library with reads of 50-bp sequencing module (Cafiso et al., 2019).

#### Tru-Seq Library Preparation and Sequencing

The samples were processed using the Illumina MiSeq, using an A PE library with reads of 150 bp and average insert size of 350/400 bp. After sequence data generation, raw reads were processed using FastQC (v.0.11.2) to assess data quality. The sequenced reads were then trimmed using Trimmomatic (v.0.33.2) to remove only sequencing adapters for PE reads. A minimum base quality of 15 (Phred scale) over a four-base sliding window was required. Only sequences with a length above 36 nucleotides were included in the downstream analysis. Similarly, only trimmed reads were included in the downstream analysis (Cafiso et al., 2019).

#### Short-Insert Library Preparation and Sequencing

The samples were processed using the Illumina MiSeq technology with an A single-end stranded library with reads of 50 bp. After sequence data generation, raw reads were processed using FastQC (v.0.11.2) to assess data quality. Reads were then trimmed using Trimmomatic (v.0.33.2) to remove sequencing adapters for single-end reads, requiring a minimum base quality of 15 (Phred scale) and a minimum read length of 15 nucleotides. Only trimmed reads were included in the downstream analysis (Cafiso et al., 2019).

#### Tru-Seq and Short-Insert Library Analysis

TS and SI RNA-seq reads were annotated on *S. aureus* NCTC 8325 RefGen (CP000253.1), as well as transcripts assembled and quantified using Rockhopper (v.2.03) (McClure et al., 2013; Tjaden, 2015), specifically designed for bacterial gene structures and transcriptomes. Analyses were run using default parameter settings with verbose output to obtain expression data. Rockhopper normalizes read counts for each sample using the upper quartile gene expression level. Starting from the *p*-values calculated according to the Anders and Huber approach, differentially expressed genes (DEGs) were assigned by computing *q*-values ≤ 0.01 on the basis of the Benjamini–Hochberg correction with a false discovery rate of <1%. In addition, Rockhopper is a versatile tool using biological replicates when available, and surrogate replicates when biological replicates for two different conditions are unavailable, considering the two conditions under investigation as surrogate replicates for each other (McClure et al., 2013).

Finally, filtering analyses were computationally carried out for sorting, first, the DEGs in the DAP^R^ strains versus their DAP^S^ parental strain and, then, a selection of only those present contemporarily in both DAP^R^ isolates showing the same expression (under-expression or over-expression) (Cafiso et al., 2019).

#### Determination of the Affected Pathways

The online tool DAVID (v.6.8) was selected to identify the affected pathways among the DEGs. The gene lists were uploaded as Official Gene Symbols of the *S. aureus* NCTC 8325 RefGen and automatically selecting the list type (Gene list) of *S. aureus*. The Functional Annotation Chart was visualized using the *p*-value threshold of 0.01 and a minimum count number of four genes. The information on the affected pathways was obtained from Kyoto Encyclopedia of Genes and Genomes (KEGG) within the analysis in DAVID, using the mentioned thresholds. The affected biological processes were obtained from the Gene Ontology (GO) Consortium within the analysis in DAVID (Cafiso et al., 2019).

#### RNA Functional Categories

Functional categories of DEGs were investigated by BLAST, PANTHER Classification System, GO Consortium, ExPASy (STRING), and KEGG pathway (Cafiso et al., 2019).

#### RNA-Sequencing Data Accession Number

RNA-seq raw reads were deposited at the Gene Expression Omnibus (GEO) database under study accession no. GSE121797 (BioProject: PRJNA498510).

#### Downstream Statistical Analysis

An heatmap of the entire set of DEGs and principal component analysis (PCA) on the five discovered DEG functional categories, in DAP^R^ MRSA versus their DAP^S^ parents, were performed by XLSTAT 2020 Excel data analysis add-on (v.2020.1.3) using default setting.

#### Real-Time qPCR

To validate RNA-seq data, real-time qPCRs were performed on the most DAP^R^-relevant transcripts (SAOUHSC_02317, SAOUHSC_00022, SAOUHSC_00486, SAOUHSC_02922, SAOUHSC_01806, SAOUHSC_01334, and SAOUHSC_00545) using RNAs of three experiments performed under the RNA-seq growth conditions. Real-time qPCRs were carried out following published protocols (Cafiso et al., 2012, 2014); the primer sequences are shown in Supplementary Table S1 and used with the following thermal profile: 95°C for 3 min; 35 cycles at 95°C for 10 s, 60°C for 30 s, and 72°C for 45 s; and a final cycle at 95°C for 1 min, 60°C for 30 s, and 95°C for 30 s. *gyr*B was the normalizer. Statistical analyses were conducted as previously described (Pfaffl et al., 2002).

### L-Lactic Acid Concentration Assay

Single strains were grown under shaking at 250 rpm in standard atmospheric conditions at 37°C and harvested in the late post-exponential growth phase. The lactate conversion was measured by enzymatic lactate oxidation into pyruvate using a D-lactic acid/L-lactic acid commercial test (R-Biopharm, Germany). L-Lactic acid concentrations were calculated using the D-lactic acid/L-lactic acid ultraviolet method according to the manufacturer’s instructions (R-Biopharm), which is based on the conversion of D-lactate or L-lactate by D-lactate dehydrogenase and L-lactate dehydrogenase, respectively, to pyruvate and NADH. The L-lactic acid concentrations were expressed in g/L. Three biological and technical replicates were performed for each strain. A statistical significance was attributed in Student’s *t*-test with *p*-values < 0.05.

## Results

### Genomics

#### Core Genome Single-Nucleotide Polymorphism Phylogeny

Pairwise cgSNP distance analysis showed a very close phylogeny of the DAP^R^ strains respect to the DAP^S^ parents. Thirty-six cgSNP differences were found between the 1A and 1C, whereas 41 cgSNPs were found between the 3A versus 3B isolated during a period of both 4 and 6 months under DAP therapy, respectively.

#### Genomic Epidemiology

The genomic epidemiology showed the membership of the 1A/C strain pair to the ST398-SCC*mec*IVa-t1939-*agr*I both harboring the plasmid pDLK1 carrying *erm*C, together with the pS194 harboring the *str* gene (Table 1) only in 1C, both conferring aminoglycoside resistance. The 3A/B strain pair was confirmed to be ST8-SCC*mec*-IVc-t008-*agr*I having the SAP063A plasmid carrying *bla*Z determining β-lactam resistance, and with the 3B having in addition the pDLK1 carrying *erm*C (Table 1).

Integrating the Center for Genomic Epidemiology and Nullarbor outputs, the strain pair resistomes (acquired AMR genes and known chromosomal SNPs conferring antimicrobial resistance) and virulomes (chromosomal and acquired virulence genes) are outlined as shown in Table 1.

#### Genome Comparison

Mauve Genome alignments of the single DAP^R^ MRSA genome versus its DAP^S^ parent showed the lack of new genetic elements associable to DAP-R. The unique additional genetic traits present in DAP^R^ MRSA strains were the two plasmids, that is, pS194 in 1C and pDLK1 in 3B.

#### SNPome and Possibly Related to Daptomycin Resistance Acquisition Non-synonymous Single-Nucleotide Polymorphisms

DAP^R/S^ strain-pair SNPome carried out versus *S. aureus* NCTC8325 RefGen recorded a variant rate of 64 in the DAP^R/S^ 1A/C strain pair with the presence of 43,720 variants in 1C and 43,947 in 1A; otherwise, a variant rate of 893 was found in 3B and 592 in 3A, with the presence of 3,157 variants and 4,762 variants, respectively.

In the DAP^R/S^ 1 A/C strain pair, according to the criteria of SnpEff tool, 0.924% mutations were putatively predicted with HI on their products; 57.118% with LI; 23.665% with MI; and 18.293% with MFI, in 1C. Among these, 23.376% were missense (M), 0.25% were nonsense (NS), and 76.374% were silent (S). In 1A, similarly, 0.978% had HI, 56.833% had LI, 23.759% had MI, and 18.431% had MFI, with 23.701% of M mutations, 0.314% of NS mutations, and 75.984% of S mutations.

**FIGURE 1:**
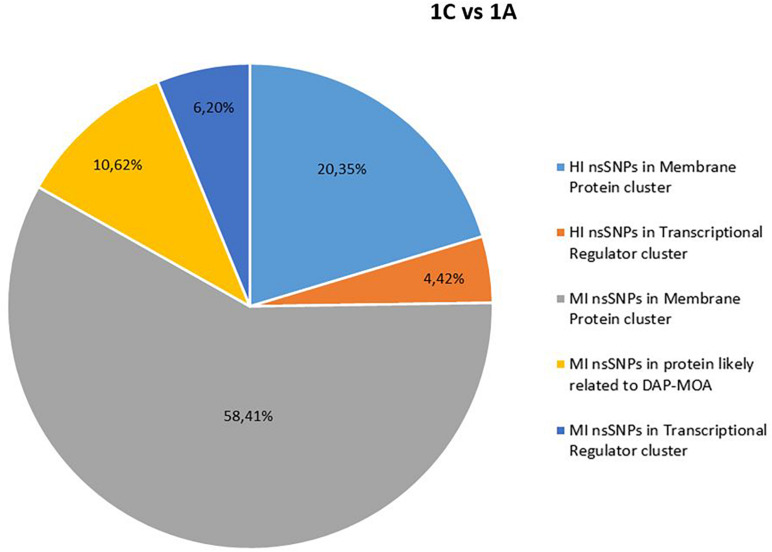
High (HI) and MI nsSNP overview of 1C versus 1A. HI, high impact; MI, moderate impact; nsSNP, non-synonymous single-nucleotide polymorphisms.

**FIGURE 2:**
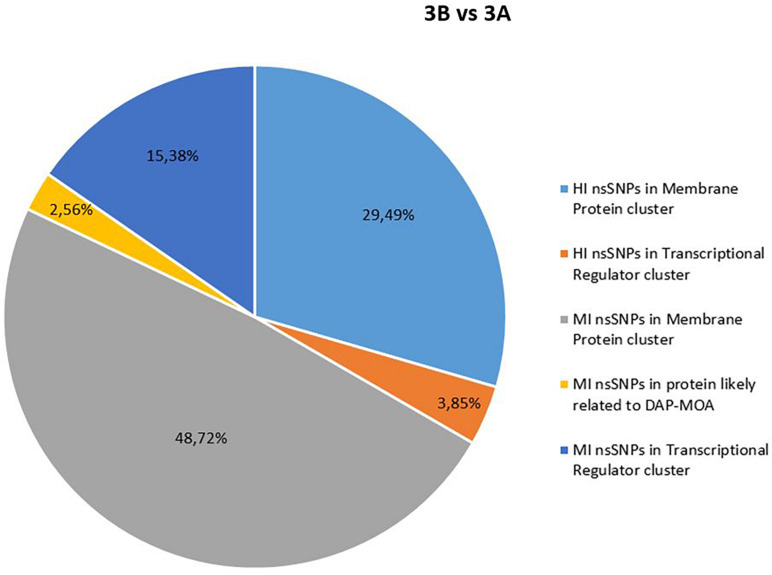
High and MI nsSNP overview of 3B versus 3A. HI, high impact; MI, moderate impact; nsSNP, non-synonymous single-nucleotide polymorphisms.

No shared HI and MI acquired nsSNPs in DAP-MOA-related targets were found in both DAP^R^ and DAP^S^ MRSA strain pairs (Figures 1, 2); however, high stringent DAVID enrichment analysis evidenced HI and/or MI DAP-MOA-related nsSNPs in the same functional protein clusters, with recovered coding genes and nsSNPs predominantly different between the two DAP^R/S^ MRSA strain pairs.

In details, HI and/or MI DAP-MOA-related nsSNPs were detected in two UP_SEQ_FEATURE clusters of targets putatively related to DAP-MOA, that is, membrane proteins (including transmembrane proteins, lipoproteins, and two-component histidine kinase systems and transporters) and transcriptional regulators. In addition, only in DAP^R^ 3B versus DAP^S^ 3A MRSA were HI DAP-MOA-related nsSNPs also recovered in the UP_SEQ_FEATURE Signal Peptide Protein cluster including gene coding proteins involved in CW turnover and teichoic acid metabolism. Furthermore, in both DAP^R^ and DAP^S^ MRSA strain pairs, MI DAP-MOA-related nsSNPs were also detected in a miscellaneous of gene coding proteins putatively related to DAP-MOA including proteins related to autolysis, cell division, peptidoglycan transport and cell shape, diacylglycerol metabolism, phosphatidylglycerol modifications (MprF), proteins involved the cardiolipin biosynthetic process (Cls1), and lipoteichoic acid biosynthesis (DltB) (Supplementary Table S2).

#### RNAome Structures

The RNAome structures, shown in TS and SI libraries, of the single DAP^R^ versus DAP^S^ strain pairs are reported in Tables 2, 3.

**TABLE 2:** RNAomes—Tru-Seq library Rockhopper summary.

	1A	1C	3A	3B
Total reads	853,091	873,327	1,010,514	1,011,410
Aligned reads	826,706 (97%)	847,504 (97%)	986,353 (98%)	979,230 (97%)
Sense	50%	50%	49%	49%
Antisense	48%	48%	46%	47%
Unannotated	1%	1%	1%	1%

	**1A and 1C**		**3A and 3B**	

5′-UTR	13		99	
3′-UTR	3		70	
Not antisense	8		11	
Antisense	19		22	
Differentially expressed genes	341		210	
Likely operons	1,057		1,122	
Multigene operons	538		547	

**TABLE 3:** RNAomes—short-insert library Rockhopper summary.

	1A	1C	3A	3B
Total reads	306,666	197,745	431,264	365,885
Aligned reads	58,942 (19%)	35,115 (18%)	155,706 (36%)	51,346 (14%)
Sense	80%	86%	53%	80%
Antisense	1%	0%	1%	0%
Unannotated	5%	7%	7%	5%

	**1A and 1C**		**3A and 3B**	

5′-UTR	10		37	
3′-UTR	5		11	
Not antisense	451		813	
Antisense	861		1,656	
Differentially expressed genes	222		413	
Likely operons	1,040		1,069	
Multigene operons	533		544	

#### DAVID Pairwise Enrichment Analysis

Integrating data obtained from the two libraries, DAVID enrichment pairwise analysis (*p*-value ≤ 0.05) (Huang et al., 2009a, b) of the over-expressed DEGs evidenced the enrichment of only the ABC transporter KEGG pathway in both DAP^R/S^ strain pairs (Supplementary Tables S3, S4). In addition, among the under-expressed DEGs, we found several enriched KEGG pathways responsible for ABC transporter, glycolysis and pyruvate metabolism, purine and propanoate metabolism, phosphotransferase system (PTS), and two-component regulatory systems. Among the TCRSs, SaeR/S (SAOUHSC_00714/SAOUHSC_00715) was found in both pairs and WalK (SAOUHSC_00021) only in the 3A/B pair (Supplementary Table S3). Diverse strain-dependent enriched over-expressed/under-expressed KEGG pathways were observed in the single 1A/C and 3A/B pair as shown in Supplementary Tables S3, S4.

#### Cross-Filtered Double-Pair Differentially Expressed Genes

**FIGURE 3:**
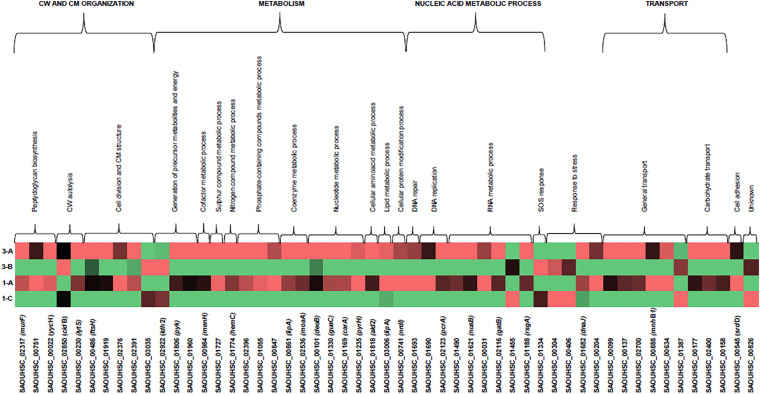
Heatmap of DEGs related to DAP-MOA and accessory traits maintained after DAP resistance onset in DAP^R^ MRSA. COG/GO cluster of genes is shown on the top of the figure, and the transcript ID and gene name are shown on the bottom. DAP^S^ (1A, 3A) and DAP^R^ MRSA (1C, 3B). DEGs, differentially expressed genes; DAP, daptomycin; COG, clusters of Orthologous Group; GO, Gene Ontology; DAP^R^, daptomycin resistant; DAP^S^, daptomycin susceptible.

**FIGURE 4:**
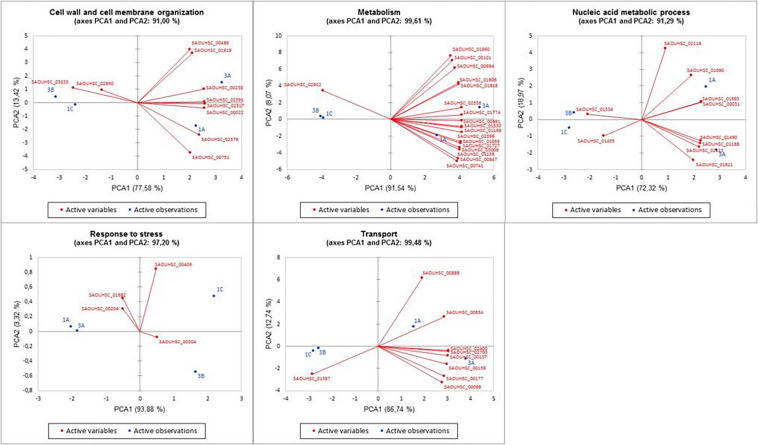
Principal component analysis of the five discovered DEG functional categories versus the DAP^R^/DAP^S^ MRSA. PCA plots show the relations between strains and genes in the different clusters. PCA, principal component analysis; DEG, differentially expressed gene; DAP^R^, daptomycin resistant; DAP^S^, daptomycin susceptible; MRSA, methicillin-resistant *Staphylococcus aureus*.

Stringently double-pair cross-refiltering of the RNA-seq data—to define the signatures exclusively present in either DAP^R^ MRSA strains or their DAP^S^ parents, and with the same expression profiling in the same library (SI or TS)—highlighted the co-occurrence of 53 statistically significant DEGs: nine over-expressed and 44 under-expressed protein-coding genes were found (Figures 3, 4 and Supplementary Table S5).

According to the GO numbers and Clusters of Orthologous Group (COG) groups, we categorized the characterizing DAP^R^ DEGs in different clusters, as follows.

##### Cell division, cell wall, and cell membrane

Three DEGs implicated in peptidoglycan biosynthesis, two DEGs for the CW autolysis, and five DEGs involved in cell division and CM structure were found.

For peptidoglycan biosynthesis, we found under-expression in the UDP-*N*-acetylmuramoyl-tripeptide-D-alanyl–D-alanine ligase *mur*F, in SAOUHSC_00751 hypothetical protein encoding gene (neighboring and consequently predicted as functionally related to the UDP-*N*-acetylenolpyruvoylglucosamine reductase MurB for the CW formation STRING_score_: 0.808), and in YycFG (WalKR) negative regulator, *yyc*H. In CW autolysis, over-expression was shown in *cid*B autolysin and under-expression in the sensor histidine kinase *lyt*S.

As concerns cell division and CM structure, under-expression was displayed in *fts*H, incorporating the PBPs into the CM, in SAOUHSC_01919, SAOUHSC_02376, and SAOUHSC_02391 uncharacterized proteins located—according to the GO Cellular Component term (GO-CC): 0016021—in the CM, whereas over-expression was recorded only in SAOUHSC_03035 integral membrane protein.

##### Metabolism

A complex network involving differential expression was shown in metabolic genes. As regards the generation of precursor metabolites and energy, L-lactate dehydrogenase-2 encoding gene *ldh*2 involved in lactic fermentation was over-expressed, whereas the pyruvate kinase gene *pyk* involved in glycolysis and the protoporphyrinogen-oxidase SAOUHSC_01960 (porphyrin biosynthesis) were under-expressed. In the cofactor metabolism, under-expression was recorded in 2-succinyl-6-hydroxy-2,4-cyclohexadiene-1-carboxylate synthase *men*H.

Regarding the compound metabolism, under-expression was found in cysteine-desulfurase SAOUHSC_01727 for sulfur-compound metabolism; in the porphobilinogen-deaminase *hem*C related to the nitrogen-compound metabolism; in SAOUHSC_02396 Cof-like hydrolase, SAOUHSC_01055 inositol-monophosphatase, and SAOUHSC_00847 ABC transporter involved in the metabolism of phosphate-containing compounds; in SAOUHSC_00861 lipoyl-synthase *lip*A for the coenzyme and cofactor metabolism; and in the molybdenum cofactor biosynthesis *moa*A.

With regard to nucleotide metabolism, phosphopentomutase *deo*B, GMP reductase *gua*C, carbamoyl-phosphate synthase small chain *car*A, and uridylate kinase *pyr*H were under-expressed. With regard to the amino acid and lipid catabolism, our data evidenced under-expression in alanine dehydrogenase *ald*2 and lipase-1 *lip*A. In the cellular protein modification process, under-expression was observed in *nrd*I coding the Class Ib ribonucleotide reductase stimulatory protein.

##### Nucleic acid metabolism

Under-expression was found in the SAOUHSC_01693 DNA-binding protein for DNA repair, and in the SAOUHSC_01690 of the DNA polymerase III complex and the ATP-dependent DNA-elicase, *pcr*A. In the RNA transcription and regulation, our data showed under-expression in DNA-binding protein HU coding gene functionally related to ClpC (STRING_score_: 0.555), in transcription anti-termination *nus*B, in SAOUHSC_00031 tRNA-dihydrouridine synthase, and in the aspartyl/glutamyl-tRNA amidotransferase subunit B *gat*B (tRNA metabolism). Examining ribosome biogenesis and maturation, we found over-expression in ATPase, whereas under-expression was found in *rsg*A (ribosome biogenesis). In SOS response, we observed over-expression in SAOUHSC_01334 hypothetical protein functionally related to LexA repressor (STRING_score_: 0.614) (DNA-damage response).

##### Stress response

Referring to the stress response, SAOUHSC_00304 luciferase-like monooxygenase (oxidative stress response) and SAOUHSC_00406 uncharacterized protein [previously annotated as poly(3-hydroxybutyrate) (PHB) depolymerase family protein] determining the use of PHB as a carbon source and energy storage in starvation conditions, were over-expressed. *dna*J (prevention of stress-denatured protein aggregation in response to hyperosmotic condition and heat shock) and the SAOUHSC_00204 globin domain protein, nitric oxide dioxygenase (nitrosative stress response), were under-expressed.

##### Transport

Under-expression was observed in SAOUHSC_00099, SAOUHSC_00137, and SAOUHSC_02700 transporters; in the SAOUHSC_00888 Na^+^/H^+^ antiporter *mnh*B1; and in SAOUHSC_00634 ABC metal ion transporter involved in cell adhesion. Over-expression was only found in SAOUHSC_01387 inorganic phosphate transmembrane transporter.

Results from carbohydrate transporters showed under-expression in SAOUHSC_00177 maltose ABC permease, in the SAOUHSC_02400 putative mannitol-specific PTS component, and in the SAOUHSC_00158 *N*-acetylmuramic acid transporter belonging to the phosphotransferase system.

##### Cell adhesion

Under-expression was shown in the serine-aspartate repeat-containing *sdr*D, a staphylococcal virulence factor.

##### Unknown function

Over-expression was observed in SAOUHSC_00826 encoding a conserved uncharacterized protein.

#### RNA-Seq Data Validation

RNA-seq data validation performed by real-time qPCR on the most DAP^R^-relevant genes confirmed the *mur*F (SAOUHSC_ 02317), *yyc*H (SAOUHSC_00022), *fts*H (SAOUHSC_00486), *ldh*2 (SAOUHSC_02922), *pyk* (SAOUHSC_01806), SOS response protein related to LexA (SAOUHSC_01334), and *sdr*D (SAOUHSC_00545) expression profiles detected in RNA-seq data in both DAP^R^ *S. aureus* and DAP^S^ parents (Supplementary Figure S5).

#### Lactic Acid Quantification Assay

**FIGURE 5:**
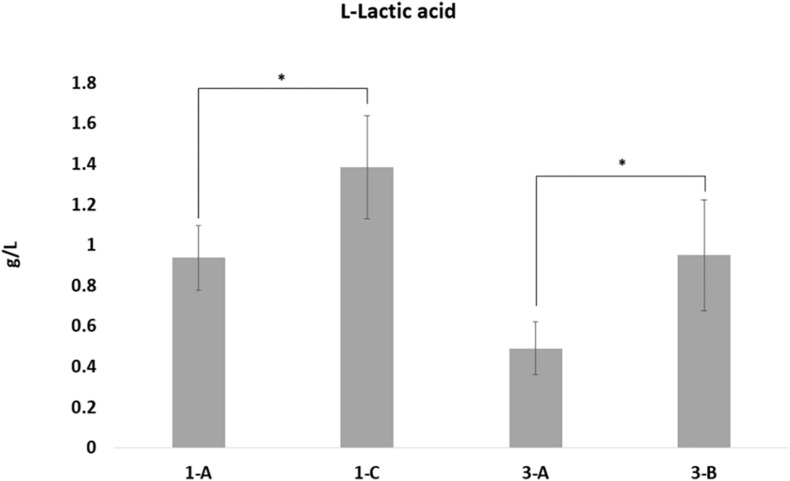
L-Lactic acid quantification. L-Lactic acid production by the four strains was calculated using the L-lactic acid ultraviolet method according to the manufacturer’s instructions (D-lactic acid/L-lactic acid kit, R-Biopharm), based on conversion of L-lactate by L-lactate dehydrogenase to pyruvate and NADH. L-Lactate production differences with *p*-values < 0.05, obtained by Student’s *t*-test, were considered statistically significant. * is for the statistical significance.

To biologically validate the bioinformatic prediction of the over-expression in L-lactate dehydrogenase-2, quantification assays of the amount of lactic acid were performed, showing a statistically significant increased concentration of L-Lactate in both DAP^R^ MRSA and their DAP^S^ parental strains (Figure 5).

## Discussion

In *S. aureus*, DAP-R is a complex and multifactorial mechanism shaped by the co-occurrence of intrinsic, acquired, and adaptive determinants that, harmonizing in concert, confer resistance. We investigated two clinical epidemiologically unrelated DAP^R/S^ MRSA strain pairs isolated from different patients in two different Italian hospitals. The two patients failed glycopeptide therapy and were then treated with DAP (Cafiso et al., 2014).

Previous investigations on the same DAP^R/S^ MRSA strain pairs characterized some traits related to DAP-R, including a dysregulation in the two key determinants of net positive surface charge, *dlt*A and *mpr*F, both during exponential and stationary growth phases; a significant increase in the D-alanylated wall teichoic acids amount correlating with DltA gain-in-function; a heightened elaboration of lysyl-phosphatidylglycerol reflecting MprF gain-in-function; an increased CM fluidity; a strain-dependent CM fatty acid perturbation due to an increase in the anteiso-branch chain species corresponding to a reduction in the major iso-branched chain and saturated fatty acids (SFAs); and a reduced susceptibility to prototypic cationic host defense peptides of platelet and leukocyte origins (Cafiso et al., 2014; Mishra et al., 2014; Boudjemaa et al., 2018).

In the present study, genome-wide CSI phylogeny and the cgSNP analysis of the same strain pairs revealed the close relation of the DAP^R/S^ MRSA isolated from the same patient under DAP therapy administered in hospital during a period of several months. Concomitantly, genomic epidemiology confirmed their previously published molecular typing (Cafiso et al., 2014) as well as plasmids and intrinsic resistance trait content; whereas acquired AMR genes and SNP profiling highlighted the acquisition of genetic traits related to the onset of additional antimicrobial resistances. In addition, virulome analysis showed a high virulence gene toxin content of the ST8 DAP^R/S^ 3A/B strain pair with respect to the ST398 DAP^R/S^ 1A/C.

Comparative genomics did not reveal newly acquired mobile genetic elements in both DAP^R^ MRSA and their DAP^S^ parents putatively related to DAP-R onset. On the contrary, *in depth*, SNPomics showed HI and MI DAP-MOA-related nsSNPs in shared functional coding-gene clusters, despite the diversity of the ST398 and ST8 genomic backgrounds with respect to the *S. aureus* NCTC8325 RefGen (proved to be the genome most closely related to both strain pairs in study).

Comparing DAP^R^ versus their DAP^S^ parents on *S. aureus* NCTC8325 RefGen, functional protein coding-gene clusters were found to be a hot spot of nsSNPs in a pairwise shared way. However, within these clusters, the detected putative HI and MI DAP-MOA-related nsSNPs in the target genes varied between the two strain pairs, indicating a strain-dependent behavior, defining two different DAP^R^ genomic backgrounds.

Our data, supported by previous findings on laboratory induced DAP^R^ MRSA of specific clones HG003, USA300-TCH1516, MSSA476, MW2, and MRSA252 mutants (Coe et al., 2019), first described and defined in two clinical DAP^R/S^ MRSA of genomic backgrounds (ST8 and ST398) strain-dependent shared and functional protein clusters as hot spots of genomic variations. In particular, membrane protein (transmembrane proteins, lipoproteins, and transporters), transcriptional regulator (Sigma-70, RpoB, RpoC, RsbU, GraX, SarR, SarU, SarX, ArlS, WalK, AgrC/A, MsrR, Msa, KdpD, SAOUHSC_02390, and SAOUHSC_00673), and cell-envelope modification (Sle1, UgtP, DltB, FmtA, LspA, Cls1, MprF, and SAOUHSC_01063) protein coding-gene clusters emerged as accumulation sites of mutational events related to the DAP-R onset, randomly occurring in the same gene. Therefore, the CM structural and the functional proteins (cell envelope, cell division, and stress response system proteins) together with several transcriptional regulators represented the cellular targets undergoing genomic gain-in changes under DAP pressure.

The late growth phase transcriptional analysis, mimicking the *in vivo* infection-site growth, outline for the first time the long-term transcriptomic fingerprinting and networks involved in the adaptation to become DAP^R^ MRSA. DAVID analysis highlighted common enriched pairwise over-expressed/under-expressed KEGG pathways in transport and TCRSs, SaeS/R, and WalKR, even though not in both strain pairs for WalK. Some differences in the enriched KEGG pathways can be attributable to strain-dependent features and different genomic background. The SaeR/S regulatory system (SAOUHSC_00714/SAOUHSC_00715) controls the production of exoproteins involved in adhesion and invasion of host cells, that is, hemolysins (*hla* and *hlb*), *coa*, Dnase, and *spa*- and CW-associated proteins (*emp*, *eap*, and *fnb*A), whereas WalK was previously related to glycopeptide resistance (Kolter et al., 1993; Shoji et al., 2011).

Transcriptomes revealed long-term imprints closely or indirectly related to the DAP-MOA and several accessory traits corroborating the multiple transcriptomic adaptations acquired with DAP-R onset, following DAP exposure and maintained by DAP^R^ MRSA.

In particular, we considered as closely related features the CW and CM structure and organization traits as well as the primary metabolism ones owing to their link to the DAP-MOA and indirectly related features whether involved in biological pathways not directly related to DAP-MOA as the survival mechanisms of bacteria, that is, oxidative stress response, reactive oxygen species (ROS) detoxification, and ABC transporters.

The cell-envelope organization and structure are a key trait closely related to the DAP-MOA showing a multilevel feedback. Changes in the cell-envelope organization and structure appear, in fact, related to the modulation of four different pathways—that is, the peptidoglycan biosynthesis, cytolysis, cell division, and CM structure—considered related to DAP-MOA targets.

In details, adaptations in CW and CM organization and changes in the profile and content of the membrane proteins—affecting the availability of these DAP targets (Pogliano et al., 2012)—can be speculated *via* a modulation of the YycFG expression by *yyc*H and membrane-protein gene under-expression. In *Bacillus subtilis*, *yyc*HI is accessory system to YycFG repressing the YycG histidine kinase function (Fukushima et al., 2008). Changes in genes modulating cell-envelope stress and maintenance, including YycFG, had been previously associated with the development of DAP^R^ in clinical and laboratory-derived DAP^R^ *S. aureus* mutants, as well as with resistance to vancomycin (Utaida et al., 2003; Gaupp et al., 2015). The YycFG was reported as a key regulator of different processes affecting CW metabolism, CM lipid homeostasis and biofilm formation, changes in membrane fluidity, and CW cross-linking compensating osmotic pressure stresses (Dubrac and Msadek, 2004; Fukushima et al., 2011; Delauné et al., 2012).

Alterations in peptidoglycan synthesis were supposed *via mur*F and SAOUHSC_00751 (functionally related to MurB) under-expression, indicating a putative decreased addition of the C-terminal D-alanyl–D-alanine dipeptide to the CW precursor muropeptide, a critical control point of peptidoglycan synthesis in *S. aureus*. The biosynthesis and attachment of the D-alanyl–D-alanine dipeptide are catalyzed by the different proteins encoded by *ddl*A and *mur*F, with the MurF attaching the dipeptide to the UDP-*N*-acetylmuramic acid (MurNAc)-tripeptide, thus completing the biosynthesis of the peptidoglycan block, the UDP-linked MurNAc-pentapeptide. The D-alanyl–D-alanine C-terminal residues are essential for reactions taking place at the CW (Sobral et al., 2006). Even though DAP is structurally related to amphomycin, and similar lipopeptides as well as peptidoglycan biosynthesis inhibitors, no experimental studies share evidence on a similar MOA (Taylor and Palmer, 2016; Gómez Casanova et al., 2017). In our opinion, this signature could represent a new putative intrinsic daptomycin/glycopeptide cross-resistance mechanism because the D-alanyl–D-alanine dipeptide also represents glycopeptide targets. This transcriptomic trait experimentally supports the association of DAP-R and glycopeptide reduced susceptibility in MRSA, as well as, for the first time, providing experimental evidence on a relationship between DAP^R^ onset and dysfunctionality in peptidoglycan biosynthesis, likely linked to a concomitance DAP inhibitor activity as reported for similar lipopeptide antimicrobials.

In addition, PBP incorporation into the CM and the quality control of cytoplasmic and integral membrane proteins could be affected through damaged-protein degradation and the protein-folding by *fts*H under-expression (Lithgow et al., 2004). Autolysis is a crucial signature of the DAP^R^ transcriptomic pathway and is closely related to the DAP-MOA, as demonstrated by *cid*B over-expression and *lyt*S under-expression. Our data showed that DAP-R could lead to an increased activity of extracellular murein hydrolases via *cid*B over-expression. CidAB and LytS/R represent the key of the bacterial lysis regulatory pathway (Groicher et al., 2000; Patton et al., 2006). Furthermore, the LytSR system has been hypothesized to function as a staphylococcal “voltmeter,” rapidly sensing Δψ changes and leading to adaptations for resistance to host defense cationic antimicrobial peptides (HDPs) (Yang et al., 2013). Because the bactericidal mechanisms of action of the HDPs, as well as DAP, involve disruption of Δψ associated with the CM (either primarily or secondarily), DAP—owing to its similarity to HDPs—could perturb the staphylococcal CM and alter transmembrane potential (ΔΨ) impacting on programmed cell death and autolysis. Under stress conditions, for example, the presence of antimicrobials, CidAB can collapse the proton motive force and allow access of autolysins to their substrate resulting in cellular lysis modulating programmed cell death (Patton et al., 2006).

Different metabolic adaptations are directly linked to the DAP-MOA characterize DAP^R^ MRSA. A fermentative metabolism appears to be the main metabolic pathway as expected in late growth phase, as demonstrated by the L-lactate dehydrogenase *ldh*2 over-expression associated with the increase in the lactic acid concentration reflecting the L-lactate dehydrogenase activity and the concomitant pyruvate kinase *pyk* under-expression. Shifting toward a fermentative metabolism leads to ATP deficiency and an alteration of membrane potential (ΔΨ) as a consequence of a decrease in the loss of oxidative phosphorylation, in agreement with previous findings showing that the succinate dehydrogenase levels, TCA enzyme, were lower in a DAP^R^ strain with respect to DAP^S^ strains (Fischer et al., 2011). *men*H under-expression (also functionally related to MurF) involved in menaquinone and phylloquinone epoxide biosynthesis was observed in DAP^R^ strains. Menaquinone is a component of the staphylococcal membrane required for correct electron transport chain (ETC) functionality; thus, the *men*H under-expression may also be related to DAP-R acquisition.

Carbohydrate and inorganic phosphate transmembrane transport represents another key point indirectly related to the DAP-MOA in DAP^R^ MRSA. In detail, our data showed a predominant under-expression of various transporter coding genes, including three genes responsible for carbohydrate transport. On the contrary, over-expression of the inorganic phosphate transmembrane transporter was observed. In particular, the over-expression of the inorganic phosphate transmembrane transporters could be implicated for its STRING predicted functionality related to the *wal*R, *arl*R, and *ssr*A master regulators involved in autolysis, biofilm formation, CW metabolism, adhesion, multidrug resistance, virulence, and the global regulation of staphylococcal virulence factors in response to environmental oxygen levels as well as repression of *agr*, *spa*, and *tst* transcription.

Accessory DAP-R fingerprints were also discovered in DAP^R^ MRSA. DAP-R alters the metabolism of amino-acid, lipid, cofactors, pyrimidine, and purine as demonstrated by the under-expression of various metabolic genes. Alterations in the pyrimidine and purine metabolic pathways have been indeed previously reported in DAP^R^ *S. aureus* (Cui et al., 2010). Looking at the nucleic acid metabolism, different genes required for DNA replication, transcription, and translation appeared differentially expressed comparing DAP^R^ versus DAP^S^ strains. The over-expression of SAOUHSC_01334—annotated as *sos*A—could indicate an SOS-response block. *sos*A appears to be LexA regulated, exhibiting an over-expression similar to that of the SOS genes (Cirz et al., 2007; Mesak et al., 2008). An SOS-response block related to hypermutator behavior is considered a key mechanism of mutational antibiotic resistance. As regards the oxidative stress response, SAOUHSC_00304 mono-oxygenase over-expression could reflect the generation of ROS (Gaupp et al., 2012). In addition, the globin domain containing protein with nitric oxide dioxygenase activity (SAOUHSC_00204) under-expression—catalyzing nitric oxide (NO) to nitrate (NO^3–^) conversion—could cause an accumulation of reactive oxidant species such as NO. All these data could indicate the involvement of oxidative stress response enzymes in DAP-R responsible for cell survival because of an increased activity of ROS detoxification. Other different heat and cold shock stress response genes were found differentially expressed in DAP^R^ *S. aureus*. In particular, *dna*J under-expression could be related to a decreased production of the chaperone protein DnaJ, involved in the response to hyper-osmotic and heat shock stress, preventing or restoring aggregation of denatured proteins. The use of different carbon sources in stress and nutrient limitation conditions were found, which could facilitate the survival of DAP^R^ strains in starvation and stress conditions *via* PHB-depolymerase family protein over-expression. As regards cell adhesion, our data showed a putative decreased adhesion ability of DAP^R^ versus DAP^S^ *S. aureus via sdr*D under-expression, although this was in contrast with previous findings (Song et al., 2013). SdrD, cell surface-associated calcium-binding protein, interacts with the extracellular matrix of higher eukaryotes.

In addition, different PCAs supported the correlation of the five distinctive functional DEG clusters with a daptomycin resistance or susceptible phenotype.

New consideration should be given for *mpr*F and *dlt*A expression. In the late post-exponential growth phase RNA-seq data, *mpr*F expression trend showed increased transcripts in 1C versus 1A and decreased in 3B versus 3A, even though not statistically significant. Even *dlt*A expression did not display statistically significant differential expression in any strain pairs; however, the expression trend displayed a decreased amount of transcripts in DAP^R^ strains in both strain pairs (data shown in files submitted to GEO). Based on these data, we hypothesize a growth-phase/strain-dependent *mpr*F expression in the late growth phase transcriptomes correlating with the increased lysyl-phosphatidylglycerol synthesis in 1C versus 1A strain pair and a decrease in 3B versus 3A strain pair described in our previously published data on stationary cultures (Mishra et al., 2014). On the contrary, a growth phase-dependent *dlt*A expression was observed in the late post-exponential growth phase in both strain pairs in agreement with other findings (Yang et al., 2009). Ultimately, although the bioinformatic cutoffs used to analyze these transcriptomic data are the standard ones, they could be too stringent both to simultaneously identify all RNAomic features characterizing DAP^R^ *S. aureus* and to fully reflect the complexity of this biological system.

## Conclusion

Our data define the complex genomic and long-term transcriptomic fingerprinting and adaptations of DAP^R^ MRSA, providing new insights into their distinctive traits focusing on targets related to DAP-MOA. Briefly, we can summarize that DAP^R^ MRSA acquired diverse genomic and transcriptomic changes to cope with and preserve bacterial cell from DAP action. CM structural/functional proteins and transcriptional regulators emerged as the cell targets related to genomic changes gained under DAP pressure. Furthermore, DAP-MOA-related transcriptomic adaptations were found in CW and CM organization, that is, peptidoglycan biosynthesis, cell division, and CM structure, as well as in the autolytic system, in primary metabolism via a shift toward fermentation, and in CM-potential perturbation. Finally, accessory traits can also impact on DAP^R^ such as multilevel amplified stress responses mainly including oxidative stress response determining cell survival due to an increased ROS detoxification and the ABC transporters.

## Data Availability Statement

The datasets generated for this study can be found in this article/Supplementary Material. WGS raw reads were deposited at Sequence Read Archive (SRA) under study accession no. SRP166981 (BioProject: PRJNA498510). RNA-seq raw reads were deposited at the Gene Expression Omnibus database (GEO) under study accession no. GSE121797 (BioProject: PRJNA498510).

## Author Contributions

VC and SSte conceived and designed the study. VC, SStr, FL, ID, and AZ performed the transcriptomics, real-time qPCR, and bioinformatics. GP contributed to the bioinformatics analysis. All authors analyzed the data and contributed to the manuscript.

## Conflict of Interest

The authors declare that the research was conducted in the absence of any commercial or financial relationships that could be construed as a potential conflict of interest.

## Footnotes

1 http://genomicepidemiology.org/

2 http://github.com/tseemann/nullarbor

3 http://david.abcc.ncifcrf.gov/

4 http://snpeff.sourceforge.net
