# Supplementary material for: Genomic and Long-Term Transcriptomic Imprints Related to the Daptomycin Mechanism of Action Occurring in Daptomycin- and Methicillin-Resistant Staphylococcus aureus Under Daptomycin Exposure
Source: Front Microbiol. 2020 Aug 14;11:1893. doi: 10.3389/fmicb.2020.01893 (PMC7456847; doi:10.3389/fmicb.2020.01893)
Supplement: Supplementary file 1 [file Data_Sheet_1.PDF]

Table S1. Primer set used in real time qPCR

Primer	Sequences (5'→3')	Fragment Size (bp)
SAOUHSC_02317-up	CGTCACAGGGTCTAATGGTAAA	224
SAOUHSC_02317-dw	CAATATCTGGTTGAGCGAGGTT	
SAOUHSC_00022-up	CAGATAGTAAGAAGAGTGAAACGAAAC	225
SAOUHSC_00022-dw	TCAAATCAGGAATCATCAAGTTATG	
SAOUHSC_00486-up	TCCAGTTGTAGTCATAGCGTTATTAT	235
SAOUHSC_00486-dw	AATCCTAGATCCCATTTCTTTGAAT	
SAOUHSC_02922-up	GTACAGTCCACAGTCCTTCACCA	200
SAOUHSC_02922-dw	TAAGAAATATCCATCAAAGCCACTAT	
SAOUHSC_01806-up	GCTGCTGGTTTATATCCTGAAGAA	253
SAOUHSC_01806-dw	CAATAATGTCTGAATGTGGACGAT	
SAOUHSC_01334-up	AGTGCGAACATTAGTGCTCACT	137
SAOUHSC_01334-dw	GCGAACACTTTCCCATCTCT	
SAOUHSC_00545-up	AGTACACAGTGGGAACAGCATCA	225
SAOUHSC_00545-dw	CATTACCTTGAGATGATACCATTTCT	
*gyr*B-up	CAACTATGAAACATTACAGCAGCGT	256
*gyr*B-dw	TGTGGCATATCCTGAGTTATATTGAAT	
