# Supplementary material for: Genomic and Long-Term Transcriptomic Imprints Related to the Daptomycin Mechanism of Action Occurring in Daptomycin- and Methicillin-Resistant Staphylococcus aureus Under Daptomycin Exposure
Source: Front Microbiol. 2020 Aug 14;11:1893. doi: 10.3389/fmicb.2020.01893 (PMC7456847; doi:10.3389/fmicb.2020.01893)
Supplement: Supplementary file 2 [file Data_Sheet_2.PDF]

Table S2. HI and MI nsNPs in DAP-MOA related targets

DAP-MOA related nsSNPs 1C *vs* 1A				
**HI nsSNPs in MP cluster (MP, LP, T)**	**HI nsSNPs in TR cluster**	**MI nsSNPs in MP cluster (MP, LP, T)**	**MI nsSNPs in DAP-MOA related Protein cluster**	**MI nsSNPs in Transcriptional Regulator cluster**
**SAOUHSC_00062** → *291E<br>**SAOUHSC_00968** → L137*<br>**SAOUHSC_00971** → Y20fs<br>**SAOUHSC_01047** → F48fs<br>**SAOUHSC_01084** → I150fs<br>**SAOUHSC_01085**(*isd*E)→ E232fs<br>**SAOUHSC_01419**(*arl*S) → S434*<br>**SAOUHSC_02009** → T395fs<br>**SAOUHSC_02661** → G6fs	**SAOUHSC_00515**(σ70-r2)→ L48*<br>**SAOUHSC_00673** → Y285*<br>**SAOUHSC_01473**(*bir*A) → R255*<br>**SAOUHSC_02301**(*rsb*U) → R260fs, F6fs<br>**SAOUHSC_02566**(*sar*R) → R82*	**SAOUHSC_00052** → V18I, K145As, D170G, S217T<br>**SAOUHSC_00053** → E110A, E143D, D164G, Q185K, T217S, K229E<br>**SAOUHSC_00055** → R84P<br>**SAOUHSC_00272** → N8T<br>**SAOUHSC_00405** → I61L, FV95YM, VY99AH, DISK102EFNE, H107Q,E240D<br>**SAOUHSC_00420** → K428T<br>**SAOUHSC_00433** → Q60K<br>**SAOUHSC_00584** → G363A<br>**SAOUHSC_00587** → V82A<br>**SAOUHSC_00591** → R151G<br>**SAOUHSC_00599** → K10T, F56S, N67K<br>**SAOUHSC_00602** → V146F<br>**SAOUHSC_00618** → K206N, C106W<br>**SAOUHSC_00683** → M210I, I175L<br>**SAOUHSC_00697** → L71R<br>**SAOUHSC_00868** → M18V, K28N<br>**SAOUHSC_00925** → S95A<br>**SAOUHSC_00970** → N94K<br>**SAOUHSC_01025** → K6Q, L378V<br>**SAOUHSC_01051** → H139Y<br>**SAOUHSC_01068** → V15F, V17I<br>**SAOUHSC_01082**(*isd*C)→ L34S<br>**SAOUHSC_01122** → T27P<br>**SAOUHSC_01131** → K66N<br>**SAOUHSC_01213** → L526F, F521V<br>**SAOUHSC_01310** → K147Q<br>**SAOUHSC_01315** → I44L<br>**SAOUHSC_01358** → S138Y, S150A<br>**SAOUHSC_01359**(*mpr*F) → S295L<br>**SAOUHSC_01380**(*nik*B) → M12I<br>**SAOUHSC_01381** → F50V<br>**SAOUHSC_01389**(*pst*S) → E178D<br>**SAOUHSC_01407** → F102V<br>**SAOUHSC_01419**(*arl*S) → S434*, L286F<br>**SAOUHSC_01628** → F35V<br>**SAOUHSC_01639** → I100L, I71L<br>**SAOUHSC_01704** → F25L<br>**SAOUHSC_01758** → S101A<br>**SAOUHSC_01761** → S152P, E134D, R2G<br>**SAOUHSC_01851** → L26P<br>**SAOUHSC_01854** → P201Q, E125A<br>**SAOUHSC_01966** → I287M<br>**SAOUHSC_01971** → H50Q<br>**SAOUHSC_02001** → I136S, I136M<br>**SAOUHSC_02008** → N11T<br>**SAOUHSC_02009** → N18T<br>**SAOUHSC_02148** → W149S<br>**SAOUHSC_02153** → I206T<br>**SAOUHSC_02247** → E241A, S326F<br>**SAOUHSC_02257** → D155E<br>**SAOUHSC_02261**(*agr*B) → I110T<br>**SAOUHSC_02264**(*agr*C) → I63T, I155L<br>**SAOUHSC_02314**(*kdp*D) → Q490P<br>**SAOUHSC_02390** → Q261P<br>**SAOUHSC_02419**(*sep*A)→ R156S<br>**SAOUHSC_02420** → S256M<br>**SAOUHSC_02459** → F23C<br>**SAOUHSC_02470** → V78F, I45M, I30M<br>**SAOUHSC_02588** → Q109R, T110S<br>**SAOUHSC_02633** → N2T<br>**SAOUHSC_02650** → T203P<br>**SAOUHSC_02687** → N128I<br>**SAOUHSC_02761** → N163I<br>**SAOUHSC_02783** → F165C<br>**SAOUHSC_02788** → E79G<br>**SAOUHSC_02789** → S12T	**SAOUHSC_00243**(NADH dehydrogenase subunit) → I27M, K31R, Q44K, E51K<br>**SAOUHSC_00427**(*sle*1) → G88S, A143S, G155S, K181N, S184R, A212T, S225N<br>**SAOUHSC_00788** → I314R SAOUHSC_00870(*dlt*B) → T140P, K317N<br>**SAOUHSC_00953**(*ugt*P) → N17I<br>**SAOUHSC_00998** (*fmt*A) → A23G, I25V, H34N, DP37EP, E49A, E58G, Va60GE, S85N, I94V, P151S, L155I, K194R, T248S, QP254KS, K257R, L260F, A265S, R275K, I278V, D280E, L288I, K324Q, A335V, K368Q<br>**SAOUHSC_01063** → I7L<br>**SAOUHSC_01082**(*isd*C) → T11M, L34S, N89K, T172A<br>**SAOUHSC_01162**(l*sp*A) → F100C, I111V<br>**SAOUHSC_01310** → R2Q, V132I, D133E, D140N, K147Q, K170Q, H174K, N197K, V300E, GP308KS, L310F, S313A, V455R, K469E, S478T<br>**SAOUHSC_01359**(*mpr*F) → A26V, D160N, V171A, F174L, F194Y, A223V, S295L, I371L, F400Y, L406I, T409I, F413L, A426V, V430A, V446I, I451L, L459I, V464Ie, L473F, I478V, R489K, L494I, I503V, M505A, K522N, D525E, N531D, L575I, I660V, E692Q, E710N<br>**SAOUHSC_02979** → S12T, N47T, I60T, K65N, F78V, P191S, E376K, A608S	**SAOUHSC_00515**(σ70-r2) → E71K, F118Y, C135R<br>**SAOUHSC_00525**(*rpo*C) → V487A, I855V<br>**SAOUHSC_00674**(*sar*X)→ L15V, H65Y, N122D,<br>**SAOUHSC_01419**(*arl*S) → Q329H, L286F, E121D<br>**SAOUHSC_02264**(*agr*C)→ V26G, I63T, I155L, Y235F, SS304TR, T329S<br>**SAOUHSC_02314**(*kdp*D)→ P67L, T70A, L147R, KN277IS, W336R, T342K, K356N, F434Y, Q490P, S492A, N759D, Y800D, E817K, Y877D, V885I<br>**SAOUHSC_02390** → A45V, V60G, V69L, I165V, I194V, Q261P, F279Y
**SNPs reversion and putative gain-in effectiveness in 1C *vs* 1A**				
**SAOUHSC_00028** → fs11L<br>**SAOUHSC_00124** → *236S<br>**SAOUHSC_00587** → fs140S<br>**SAOUHSC_00646** → *11L<br>**SAOUHSC_00660** → *320L<br>**SAOUHSC_00762** → *294L<br>**SAOUHSC_01113** → fs33F<br>**SAOUHSC_01489** → fs44K<br>**SAOUHSC_02151** → fs245D<br>**SAOUHSC_02153** → fs33D<br>**SAOUHSC_02312**(*kdp*A)→ *15Y<br>**SAOUHSC_02777** → *33E<br>**SAOUHSC_02818** → fs277I<br>**SAOUHSC_03000** → *20L				

DAP-MOA related nsSNPs 3B *vs* 3A				
**HI nsSNPs in MP cluster (MP, LP, T)**	**HI nsSNPs in TR cluster**	**MI nsSNPs in MP cluster (MP, LP, T)**	**MI nsSNPs in DAP-MOA related Protein cluster**	**MI nsSNPs in Transcriptional Regulator cluster**
**SAOUHSC_00672** → R188*<br>**SAOUHSC_00916** → Y133*<br>**SAOUHSC_01402**(*msa*) → F58fs<br><br>**SNPs reversion and putative gain in effectiveness in 3B strain *vs* 3A**<br><br>**SAOUHSC_00091** → *104Y<br>**SAOUHSC_00172** → *234Y<br>**SAOUHSC_00586** → fs50K<br>**SAOUHSC_00618** → fs118L<br>**SAOUHSC_00668** → *207L<br>**SAOUHSC_00731** → fs140Q<br>**SAOUHSC_00911** → fs603K, E605*<br>**SAOUHSC_00929** → *43E<br>**SAOUHSC_00998**(*fmt*A) → fs302I<br>**SAOUHSC_01219**(*lyt*N) → fs128E<br>**SAOUHSC_01238** → Y261*<br>**SAOUHSC_01382** → *60L<br>**SAOUHSC_00731** → fs140Q<br>**SAOUHSC_01423** → K205*<br>**SAOUHSC_01427** → fs460E<br>**SAOUHSC_01448**(*nor*B) → fs258L<br>**SAOUHSC_01607** → fs21K<br>**SAOUHSC_01739**(*lyt*H) → E292*<br>**SAOUHSC_01769** → fs273Q<br>**SAOUHSC_01902** → fs55I	**SAOUHSC_00913** → *290K<br>**SAOUHSC_01402**(*msa*) → F58fs<br>**SAOUHSC_02264**(*agr*C) → L30*	**SAOUHSC_00133** → D197V<br>**SAOUHSC_00402** → G100E, H120Y,<br>**SAOUHSC_00405** → F139L<br>**SAOUHSC_00428** → F62L<br>**SAOUHSC_00585** → F68L, E69G<br>**SAOUHSC_00586** → L81R, Y120S<br>**SAOUHSC_00588** → R15K, I140L<br>**SAOUHSC_00599** → F153I<br>**SAOUHSC_00618** → Y86S<br>**SAOUHSC_00691**(uptP) → K289N<br>**SAOUHSC_00808** → R128S, K121Q,<br>S51R, E48D, H32Q<br>**SAOUHSC_00810** → I44L<br>**SAOUHSC_00916** → Y11S, K61Q<br>A114T, I144L, L208F, K255E<br>**SAOUHSC_00931** → I211S<br>**SAOUHSC_00968** → D34N, K50E<br>K183R, W215S<br>**SAOUHSC_00970** → D6E<br>**SAOUHSC_01084** → K155N<br>**SAOUHSC_01113** → S79R<br>**SAOUHSC_01122** → T31P<br>**SAOUHSC_01162**(*lsp*A)→ F66V, F66C<br>**SAOUHSC_01275** → E269A<br>**SAOUHSC_01312** → I13R, L33S<br>**SAOUHSC_01358** → N127H<br>**SAOUHSC_01377**(*nik*E)→ F210L<br>**SAOUHSC_01381** → F54V<br>**SAOUHSC_01382** → L10V<br>**SAOUHSC_01456** → L435V<br>**SAOUHSC_01462**(*gps*B) → F112S, N81K, N81T<br>**SAOUHSC_01484** → S6L<br>**SAOUHSC_01505** → I165M<br>**SAOUHSC_01639** → K94N<br>**SAOUHSC_01854** → E252K, A229E<br>**SAOUHSC_02247** → Y9S<br>**SAOUHSC_02588** → Y159F<br>**SAOUHSC_02668** → K47N<br>**SAOUHSC_02796** → M22L, I11S<br>**SAOUHSC_A02795** → H18R<br>**SAOUHSC_A02856** → T22P	**SAOUHSC_01063**→ K2E<br>**SAOUHSC_01359**(*mprF*) → W424R	**SAOUHSC_00021**(*wal*K) → A189D<br>**SAOUHSC_00524**(*rpo*B) → H434Y, H459Y<br>**SAOUHSC_00664**(*gra*X)→ D95V<br>**SAOUHSC_00673** → K319N, E356D<br>**SAOUHSC_00915** → D68E, W69G<br>F88C, G91R<br>**SAOUHSC_01361(***msr*R**)** → L42V<br>**SAOUHSC_01402**(*msa*) → Y67D, F47L<br>**SAOUHSC_02264**(*agr*C) → K121T<br>**SAOUHSC_02265**(*agr*A)→ A143T<br>**SAOUHSC_02301**(*rsb*U) → P230Q<br>**SAOUHSC_02566**(*sar*R) → P62T<br>**SAOUHSC_02800**(*sar*U)→ R243G

Legend: * stop codon; frameshift (fs); Transmembrane Protein (MP), Lipoproteins (LP), Transporters (T), Transcriptional regulators (TR)
