# Supplementary material for: Genomic and Long-Term Transcriptomic Imprints Related to the Daptomycin Mechanism of Action Occurring in Daptomycin- and Methicillin-Resistant Staphylococcus aureus Under Daptomycin Exposure
Source: Front Microbiol. 2020 Aug 14;11:1893. doi: 10.3389/fmicb.2020.01893 (PMC7456847; doi:10.3389/fmicb.2020.01893)
Supplement: Supplementary file 3 [file Data_Sheet_3.PDF]

Table S3. DAVID Enrichment Analysis on over-expressed DEGs (p-value ≤0.05)

1A/1C pair				3A/3B pair			
**KEGG-Pathway**	**Counted Gene n°**	**Genes**	**FE**	**KEGG-Pathway**	**Counted Gene n°**	**Genes**	**FE**
**ABC transporters**	13	SAOUHSC_00731, SAOUHSC_02546, SAOUHSC_00692, SAOUHSC_00136, SAOUHSC_02397, SAOUHSC_00423, SAOUHSC_00105, SAOUHSC_01385, SAOUHSC_01386, SAOUHSC_01387, SAOUHSC_00613, SAOUHSC_00749, SAOUHSC_02742	10,7	**ABC-transporters**	10	SAOUHSC_01389, SAOUHSC_00732, SAOUHSC_02003, SAOUHSC_00692, SAOUHSC_00240, SAOUHSC_00927, SAOUHSC_00842, SAOUHSC_00844, SAOUHSC_01387,SAOUHSC_02743	14,2
Two-component system	8	SAOUHSC_02099, SAOUHSC_02098, SAOUHSC_02262, SAOUHSC_01799, SAOUHSC_00558, SAOUHSC_02677, SAOUHSC_00233, SAOUHSC_00336	14,8	Glycine, serine and threonine metabolism	4	SAOUHSC_02723, SAOUHSC_01321, SAOUHSC_01320, SAOUHSC_01307	24,0
Valine, leucine and isoleucine degradation	5	SAOUHSC_00132, SAOUHSC_02860, SAOUHSC_00558, SAOUHSC_01613, SAOUHSC_00336	31,1				
Terpenoid backbone biosynthesis	5	SAOUHSC_00579, SAOUHSC_01618, SAOUHSC_02860, SAOUHSC_00558, SAOUHSC_00336	31,1				
Pyruvate metabolism	5	SAOUHSC_02849, SAOUHSC_00132, SAOUHSC_00558, SAOUHSC_02922, SAOUHSC_00336	12,4				
Glyoxylate and dicarboxylate metabolism	4	SAOUHSC_01802, SAOUHSC_01845, SAOUHSC_02582, SAOUHSC_00142	38,7				
Base excision repair	4	SAOUHSC_01658, SAOUHSC_01768, SAOUHSC_01796, SAOUHSC_01469	34,8				
Butanoate metabolism	4	SAOUHSC_00132, SAOUHSC_02860, SAOUHSC_00558, SAOUHSC_00336	16,6				
Propanoate metabolism	4	SAOUHSC_00132, SAOUHSC_00558, SAOUHSC_02922, SAOUHSC_00336	15,8				
Protein export	4	SAOUHSC_02327, SAOUHSC_00903, SAOUHSC_00902, SAOUHSC_00328	23,2				
