# Supplementary material for: Genomic and Long-Term Transcriptomic Imprints Related to the Daptomycin Mechanism of Action Occurring in Daptomycin- and Methicillin-Resistant Staphylococcus aureus Under Daptomycin Exposure
Source: Front Microbiol. 2020 Aug 14;11:1893. doi: 10.3389/fmicb.2020.01893 (PMC7456847; doi:10.3389/fmicb.2020.01893)
Supplement: Supplementary file 4 [file Data_Sheet_4.PDF]

Table S4. DAVID Enrichment Analysis on under-expressed DEGs (p-value≤0.05)

1A/1C pair

KEGG-Pathway	Counted Gene n°	Genes	FE
ABC transporters	10	SAOUHSC_01021,SAOUHSC_00652, SAOUHSC_00924,SAOUHSC_00138, SAOUHSC_02547,SAOUHSC_00137, SAOUHSC_02546, SAOUHSC_00927, SAOUHSC_02427, SAOUHSC_00177	6,8
Pyruvate metabolism	8	SAOUHSC_02142,SAOUHSC_01806, SAOUHSC_01041,SAOUHSC_02647, SAOUHSC_01042,SAOUHSC_01710, SAOUHSC_01624, SAOUHSC_01820	16,5
Arginine and proline metabolism	6	SAOUHSC_02142, SAOUHSC_01128, SAOUHSC_00898, SAOUHSC_01129, SAOUHSC_00899, SAOUHSC_00150	16,7
Oxidative phosphorylation	5	SAOUHSC_02140, SAOUHSC_01031, SAOUHSC_02343, SAOUHSC_02345, SAOUHSC_00412	16,4
Glycolysis Gluconeogenesis	5	SAOUHSC_02142, SAOUHSC_00608, SAOUHSC_01806, SAOUHSC_01041, SAOUHSC_01042	10,3
Two-component system	5	SAOUHSC_02681, SAOUHSC_01800, SAOUHSC_01586, SAOUHSC_00230, SAOUHSC_00714	7,7
Purine metabolism	5	SAOUHSC_01806, SAOUHSC_01129, SAOUHSC_00101, SAOUHSC_01330, SAOUHSC_00942	7,0
Alanine, aspartate and glutamate metabolism	4	SAOUHSC_01818, SAOUHSC_01169, SAOUHSC_00898, SAOUHSC_00899	15,2
Propanoate metabolism	4	SAOUHSC_02142, SAOUHSC_01710, SAOUHSC_01624, SAOUHSC_01820	13,2
Phosphotransferase system (PTS)	4	SAOUHSC_00158, SAOUHSC_02402, SAOUHSC_02400, SAOUHSC_02848	12,1

3A/3B pair

KEGG-Pathway	Counted Gene n°	Genes	FE
Purine metabolism	12	SAOUHSC_02485, SAOUHSC_01806, SAOUHSC_02965, SAOUHSC_01742, SAOUHSC_02369, SAOUHSC_01743, SAOUHSC_00101, SAOUHSC_01330, SAOUHSC_00097, SAOUHSC_01015, SAOUHSC_01811, SAOUHSC_00472	12,6
Pyruvate metabolism	8	SAOUHSC_01806, SAOUHSC_02849, SAOUHSC_01809, SAOUHSC_01043, SAOUHSC_01808, SAOUHSC_01820, SAOUHSC_01910, SAOUHSC_01623	12,5
Pyrimidine metabolism	7	SAOUHSC_02485, SAOUHSC_01169, SAOUHSC_02369, SAOUHSC_02377, SAOUHSC_00097, SAOUHSC_00785, SAOUHSC_01811	8,7
ABC transporters	7	SAOUHSC_00641, SAOUHSC_02152, SAOUHSC_00137, SAOUHSC_00136, SAOUHSC_02397, SAOUHSC_00424, SAOUHSC_00177	3,6
Fatty acid biosynthesis	6	SAOUHSC_01198, SAOUHSC_01199, SAOUHSC_01809, SAOUHSC_01808, SAOUHSC_00921, SAOUHSC_01623	25,2
Phosphotransferase system (PTS)	6	SAOUHSC_00312, SAOUHSC_00158, SAOUHSC_00235, SAOUHSC_00708, SAOUHSC_00437, SAOUHSC_02400	13,6
Glycolysis Gluconeogenesis	6	SAOUHSC_01806, SAOUHSC_01043, SAOUHSC_01910, SAOUHSC_00798, SAOUHSC_01646, SAOUHSC_02822	9,4
Two-component system	6	SAOUHSC_00021, SAOUHSC_00715, SAOUHSC_01065, SAOUHSC_00230, SAOUHSC_02678, SAOUHSC_00714	7,0
Pentose phosphate pathway	5	SAOUHSC_00100, SAOUHSC_00101, SAOUHSC_02612, SAOUHSC_00472, SAOUHSC_02822	16,1
Peptidoglycan biosynthesis	5	SAOUHSC_02317, SAOUHSC_01373, SAOUHSC_01374, SAOUHSC_01467, SAOUHSC_02527	13,6
Amino sugar and nucleotide sugar metabolism	5	SAOUHSC_02976, SAOUHSC_00298, SAOUHSC_02352, SAOUHSC_01646, SAOUHSC_00088	9,1
Valine, leucine and isoleucine degradation	4	SAOUHSC_01043, SAOUHSC_01613, SAOUHSC_01611, SAOUHSC_00536	15,6
Fructose and mannose metabolism	4	SAOUHSC_02976, SAOUHSC_00708, SAOUHSC_02400, SAOUHSC_02822	12,8
Mismatch repair	4	SAOUHSC_01099, SAOUHSC_02123, SAOUHSC_02122, SAOUHSC_01811	12,8
Citrate cycle (TCA cycle)	4	SAOUHSC_01416, SAOUHSC_01802, SAOUHSC_01043, SAOUHSC_01910	10,9
Propanoate metabolism	4	SAOUHSC_01809, SAOUHSC_01808, SAOUHSC_01820, SAOUHSC_01623	9,9
Glycine, serine and threonine metabolism	4	SAOUHSC_02839, SAOUHSC_01043, SAOUHSC_02840, SAOUHSC_02932	8,7
