# Supplementary material for: Genomic and Long-Term Transcriptomic Imprints Related to the Daptomycin Mechanism of Action Occurring in Daptomycin- and Methicillin-Resistant Staphylococcus aureus Under Daptomycin Exposure
Source: Front Microbiol. 2020 Aug 14;11:1893. doi: 10.3389/fmicb.2020.01893 (PMC7456847; doi:10.3389/fmicb.2020.01893)
Supplement: Supplementary file 5 [file Data_Sheet_5.PDF]

Table S5. DEGs in DAP^{R} *vs* DAP^{S} MRSA

					Expression of RNA-seq data annotated on NCTC 8325 *S.aureus* (*q-value* ≤0,01)					
**Regulation**	***S.aureus* NCTC 8325 Locus Tag**	**Description**	**COG[a]**	**GO number[b]**	**1C**	**1A**	***q*Value***	**3B**	**3A**	***q*Value***
		**Cell Wall and Cell Membrane Organization**								
		**Peptidoglycan Biosynthesis**								
↓	SAOUHSC_02317	UDP-N-acetylmuramoyl-tripeptide--D-alanyl-D-alanine ligase MurF	M	GO:0009252	0	34	4,483E-4	0	39	1,11E-12
↓	SAOUHSC_00751	Uncharacterized protein functionally related to *mur*B	S	GO:0009987	0	53	3,56E-06	0	21	3,56E-06
↓	SAOUHSC_00022	Regulatory protein YycH	S	-	0	49	0	0	52	0
		**Cytolysis**								
↑	SAOUHSC_02850	Holin-like protein CidB	M	GO:0019835	49	0	0	277	54	1,94E-3
↓	SAOUHSC_00230	Sensor histidine kinase LytS	T	GO:0071555	0	14	1,60E-19	0	21	0
		**Cell Wall Division and Cell Membrane Structure**								
↓	SAOUHSC_00486	ATP-dependent zinc metalloprotease FtsH	O	GO:0006508	25	101	7,41E-3	54	422	8,53E-3
↓	SAOUHSC_01919	Integral membrane protein	-	-	0	95	0	0	463	0
↓	SAOUHSC_02376	Membrane protein	S	-	0	221	0	0	143	1,57E-08
↓	SAOUHSC_02391	Integral membrane uncharacterized protein	-	-	0	3708	0	333	4282	6,07E-4
↑	SAOUHSC_03035	Membrane spanning protein	S	-	21	0	0	40	0	0
		**Metabolism**								
		**Generation of Precursor Metabolites and Energy**								
↑	SAOUHSC_02922	L-lactate dehydrogenase 2 Ldh2	C	GO:0006096	28	0	0	42	1	1,04E-07
↓	SAOUHSC_01806	Pyruvate kinase Pyk	G	GO:0006096	0	40	0	0	98	0
↓	SAOUHSC_01960	Protoporphyrinogen oxidase	H	GO:0022904	0	44	5,81E-15	0	264	0
		**Cofactor Metabolic Process**								
↓	SAOUHSC_00984	2-succinyl-6-hydroxy-2,4-cyclohexadiene-1-carboxylate synthase MenH	I	GO:0006732	0	35	0	0	124	1,32E-05
		**Sulphur Compound Metabolic Process**								
↓	SAOUHSC_01727	Cysteine desulfurase	E	GO:0006790	0	56	0	0	57	0
		**Nitrogen Compound Metabolic Processes**								
↓	SAOUHSC_01774	Porphobilinogen deaminase HemC	H	GO:0006778	0	95	0	0	138	0
		**Phosphate-Containing Compounds Metabolic Process**								
↓	SAOUHSC_02396	Cof-like hydrolase	R	GO:0006796	0	60	3,58E-07	0	70	0
↓	SAOUHSC_01055	Inositol monophosphatase	G	GO:0006796	0	78	0	0	81	0
↓	SAOUHSC_00847	ABC transporter	O	GO:0006796	0	86	0	0	72	4,95E-16
		**Coenzyme Metabolic Process**								
↓	SAOUHSC_00861	Lipoyl synthase LipA	H	GO:0009107	0	69	0	0	93	9,17E-39
↓	SAOUHSC_02536	Molybdenum cofactor biosynthesis protein A MoaA	H	GO:0006777	0	58	5,28E-13	0	96	4,97E-49
		**Nucleotide Metabolic Process**								
↓	SAOUHSC_00101	Phosphopentomutase DeoB	G	GO:0009117	0	289	0	65	1256	6,75E-07
↓	SAOUHSC_01330	Guanosine monophosphate reductaseGuaC	F	GO:0006163	0	210	0	0	262	0
↓	SAOUHSC_01169	Carbamoyl-phosphate synthase small chain CarA	E, F	GO:0006221	0	26	1,12E-29	0	32	0
↓	SAOUHSC_01235	Uridylate kinase PyrH	F	GO:0006221	0	79	0	0	74	1,11E-12
		**Cellular Aminoacid Metabolic Process**								
↓	SAOUHSC_01818	Alanine dehydrogenase 2 Ald2	E	GO:0009063	0	56	0	0	132	0
		**Lipid Metabolic Process**								
↓	SAOUHSC_03006	Lipase 1 LipA	R	GO:0016042	0	28	2,82E-14	0	27	1,88E-14
		**Cellular Protein Modification Process**								
↓	SAOUHSC_00741	Ribonucleotide reductase stimulatory protein NrdI	F	GO:0006464	0	265	0	0	214	0
		**Nucleic Acid Metabolic Process**								
		**DNA Repair**								
↓	SAOUHSC_01693	DNA-binding protein	L	GO:0006281	0	78	5,02E-08	0	59	6,93E-3
		**DNA Replication**								
↓	SAOUHSC_01690	DNA polymerase III subunitδ	L	GO:0006260	0	126	0	0	46	3,86E-06
↓	SAOUHSC_02123	ATP-dependent DNA helicase PcrA	L	GO:0006260	0	23	3,58E-07	0	44	0
		**RNA Metabolic Process**								
↓	SAOUHSC_01490	DNA-binding protein HU	L	GO:1903506	0	249	1,75E-18	0	411	0
↓	SAOUHSC_01621	Transcription antitermination protein NusB	K	GO:1903506	0	42	4,39E-06	0	126	0
↓	SAOUHSC_00031	tRNA-dihydrouridine synthase	-	GO:0006399	0	220	0	0	168	0
↓	SAOUHSC_02116	Aspartyl/glutamyl-tRNA amidotransferase subunit B GatB	J	GO:0006399	0	37	5,71E-08	0	70	0
↑	SAOUHSC_01455	GTPase	-	GO:0006399	45	0	0	11	0	0

↓	SAOUHSC_01188	Ribosome biogenesis GTPase RsgA	R	GO:0042254	0	56	1,57E-05	0	98	9,17E-39
↑	SAOUHSC_01334	Presumptive SOS response gene functionally related to LexA	-	-	61	0	0	134	0	0
		**RESPONSE TO STRESS**								
↑	SAOUHSC_00304	Monooxygenase	C	GO:0006979	63	0	2,47E-12	57	0	0
↑	SAOUHSC_00406	PHB depolymerase	-	GO:0042594	53	0	0	28	0	0
↓	SAOUHSC_01682	Chaperone protein DnaJ	O	GO:0009408	16	144	9,67E-05	0	180	0
↓	SAOUHSC_00204	Globin domain containing protein - Nitric oxide dioxygenase	C	GO:0071500	0	55	0	0	35	6,93E-3
		**TRANSPORT**								
↓	SAOUHSC_00099	Transporter	G	GO:0055085	0	90	0	0	370	0
↓	SAOUHSC_00137	Transporter	P	GO:0006810	0	26	0	0	48	0
↓	SAOUHSC_02700	Transporter	-	GO:0055085	0	19	0	0	32	0
↓	SAOUHSC_00888	Na+/H+ antiporter subunit B1 MnhB1	P	GO:0006812	0	115	0	0	40	0
↓	SAOUHSC_00634	ABC transporter	P	GO:0030001	0	103	0	0	96	0
↑	SAOUHSC_01387	Inorganic phosphate transmembrane transporter	P	GO:0006817	68	0	0	48	2	1,55E-07
		**Carbohydrate Transport**								
↓	SAOUHSC_00177	Maltose ABC transporter permease	G	GO:0008643	0	45	0	0	142	0
↓	SAOUHSC_02400	PTS system, mannitol-specific component	G	GO:0008643	0	40	5,81E-15	0	66	0
↓	SAOUHSC_00158	PTS system N-acetylmuramic acid-specific component	G	GO:0008643	0	33	1,57E-05	0	73	0
		**CELL ADHESION**								
↓	SAOUHSC_00545	Serine-aspartate repeat-containing protein D - SdrD	-	GO:0007155	0	99	0	0	30	0
		**UNKNOWN**								
↑	SAOUHSC_00826	Conserved uncharacterized protein	S	-	38	0	0	19	0	1,626E-05

Legend:

↑ up-regulated mRNAs in DAP^R *vs* DAP^S
↓ down-regulated mRNAs in DAP^R *vs* DAP^S
a – COG categories: C energy production and conversion, E amino acid metabolism and transport, F nucleotide metabolism and transport, G carbohydrate metabolism and transport, H coenzyme metabolism, I lipid metabolism, J translation, K transcription, L replication and repair, M cell wall/membrane/envelope biogenesis, O post-translational modification, protein turnover, chaperone functions, P Inorganic ion transport and metabolism, R general functional prediction only, S function unknown, T signal transduction.
b – Gene Ontology numbers refer only to the biological process
**q*-value=control the false discovery rate using the Benjamini-Hochberg procedure. Significant *q*-value ≤ 0,01.
