# Supplementary figures and images for: Genomic and Long-Term Transcriptomic Imprints Related to the Daptomycin Mechanism of Action Occurring in Daptomycin- and Methicillin-Resistant Staphylococcus aureus Under Daptomycin Exposure
Source: Front Microbiol. 2020 Aug 14;11:1893. doi: 10.3389/fmicb.2020.01893 (PMC7456847; doi:10.3389/fmicb.2020.01893)

**Figure S1. Contigs Coverage Histograms**

a) 1A sample

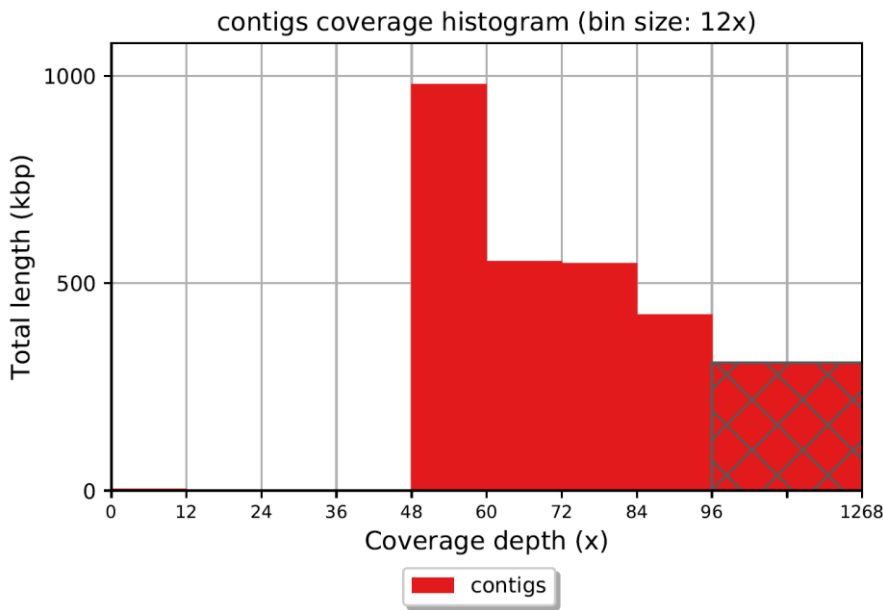

b) 1C sample

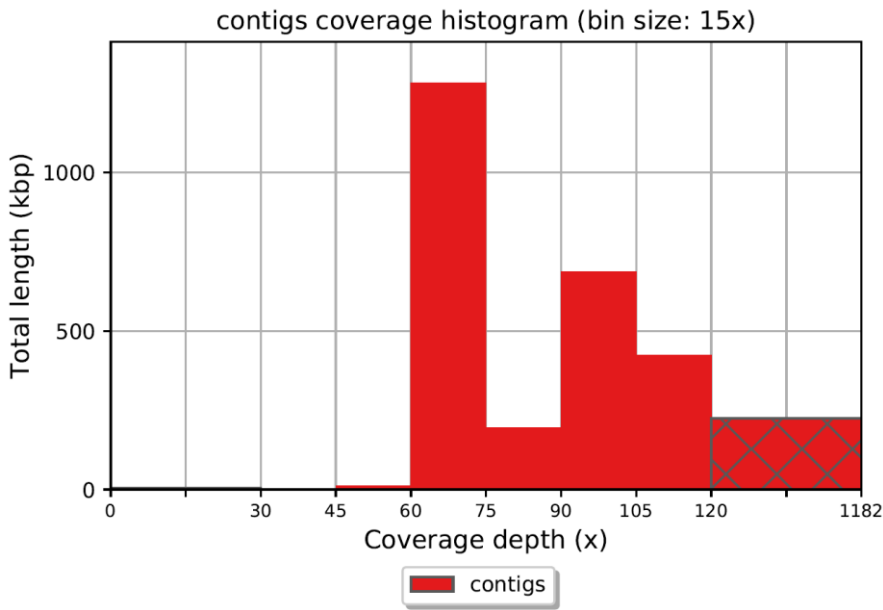

c) 3A sample

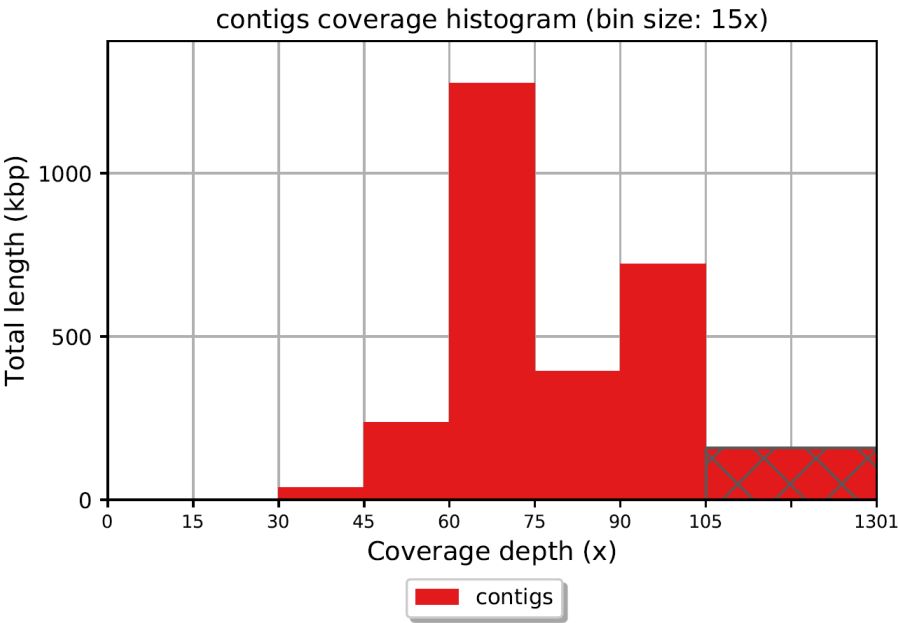

d) 3B sample

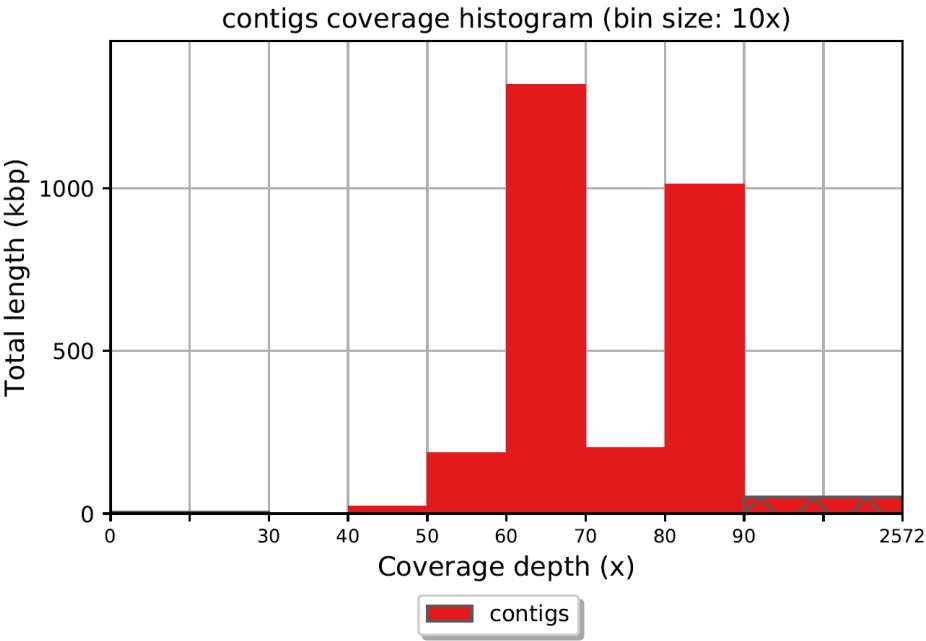

Supplement: Supplementary file 6 [file Data_Sheet_6.PDF]

Figure S3. CSI Phylogeny Tree

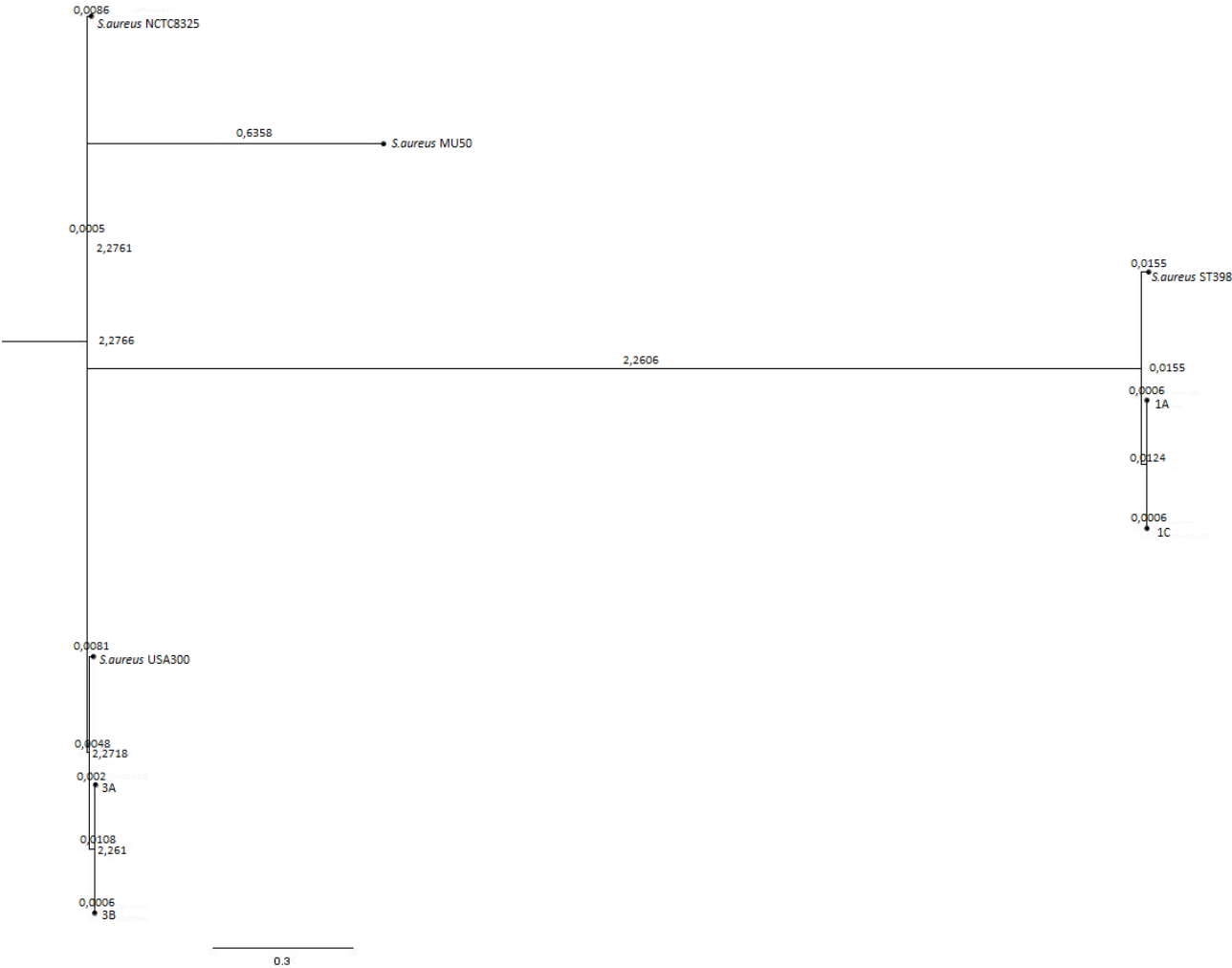

Supplement: Supplementary file 8 [file Data_Sheet_8.PDF]

Figure S5. real time qPCR validation of RNA-seq data in DAP<sup>R</sup> vs DAP<sup>S</sup> *S.aureus* parents

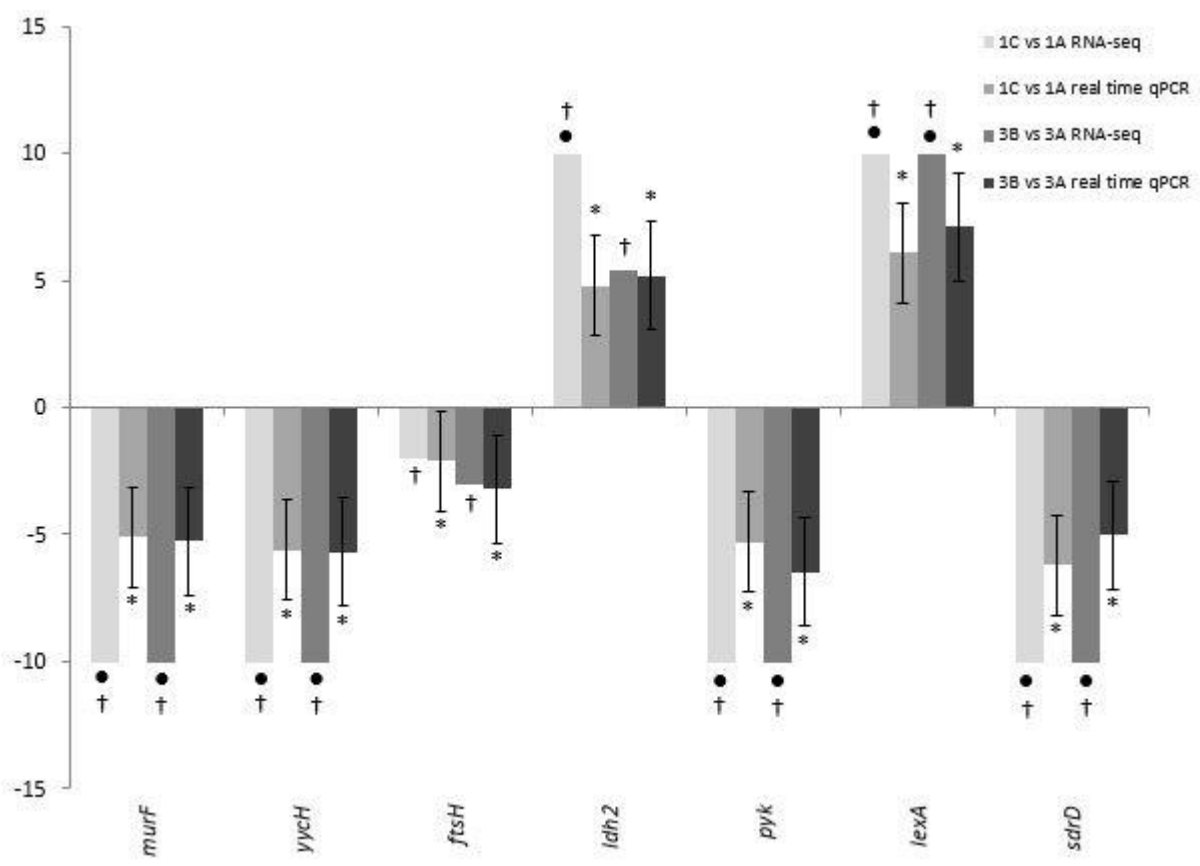

Supplement: Supplementary file 10 [file Data_Sheet_10.PDF]
