# Supplementary material for: Genomic and Long-Term Transcriptomic Imprints Related to the Daptomycin Mechanism of Action Occurring in Daptomycin- and Methicillin-Resistant Staphylococcus aureus Under Daptomycin Exposure
Source: Front Microbiol. 2020 Aug 14;11:1893. doi: 10.3389/fmicb.2020.01893 (PMC7456847; doi:10.3389/fmicb.2020.01893)
Supplement: Supplementary file 7 [file Data_Sheet_7.PDF]

Figure S2. ***de novo*** **Genome Assembly Reports**

a) 1A sample

	contigs
# contigs (>= 0 bp)	90
# contigs (>= 1000 bp)	41
# contigs (>= 5000 bp)	30
# contigs (>= 10000 bp)	29
# contigs (>= 25000 bp)	22
# contigs (>= 50000 bp)	18
Total length (>= 0 bp)	2827020
Total length (>= 1000 bp)	2811194
Total length (>= 5000 bp)	2785929
Total length (>= 10000 bp)	2780468
Total length (>= 25000 bp)	2651167
Total length (>= 50000 bp)	2497607
# contigs	65
Largest contig	414000
Total length	2822550
GC (%)	32.88
N50	168900
N75	72234
L50	6
L75	12
# N's per 100 kbp	3.54

b) 1C sample

	contigs
# contigs (>= 0 bp)	100
# contigs (>= 1000 bp)	41
# contigs (>= 5000 bp)	29
# contigs (>= 10000 bp)	28
# contigs (>= 25000 bp)	23
# contigs (>= 50000 bp)	18
Total length (>= 0 bp)	2839603
Total length (>= 1000 bp)	2822899
Total length (>= 5000 bp)	2793751
Total length (>= 10000 bp)	2788284
Total length (>= 25000 bp)	2698533
Total length (>= 50000 bp)	2497950
# contigs	62
Largest contig	352841
Total length	2832544
GC (%)	32.87
N50	168903
N75	70827
L50	6
L75	13
# N's per 100 kbp	3.53

c) 3A sample

	contigs
# contigs (>= 0 bp)	194
# contigs (>= 1000 bp)	34
# contigs (>= 5000 bp)	27
# contigs (>= 10000 bp)	24
# contigs (>= 25000 bp)	20
# contigs (>= 50000 bp)	15
Total length (>= 0 bp)	2855235
Total length (>= 1000 bp)	2819164
Total length (>= 5000 bp)	2807551
Total length (>= 10000 bp)	2787213
Total length (>= 25000 bp)	2709662
Total length (>= 50000 bp)	2526357
# contigs	56
Largest contig	363234
Total length	2830549
GC (%)	32.69
N50	262831
N75	109483
L50	5
L75	10
# N's per 100 kbp	0.00

d) 3B sample

	contigs
# contigs (>= 0 bp)	55
# contigs (>= 1000 bp)	22
# contigs (>= 5000 bp)	15
# contigs (>= 10000 bp)	13
# contigs (>= 25000 bp)	12
# contigs (>= 50000 bp)	10
Total length (>= 0 bp)	2804142
Total length (>= 1000 bp)	2793011
Total length (>= 5000 bp)	2775341
Total length (>= 10000 bp)	2760863
Total length (>= 25000 bp)	2741492
Total length (>= 50000 bp)	2674536
# contigs	41
Largest contig	731906
Total length	2801674
GC (%)	32.64
N50	402454
N75	131275
L50	3
L75	5
# N's per 100 kbp	3.57
