# Supplementary material for: Genomic and Long-Term Transcriptomic Imprints Related to the Daptomycin Mechanism of Action Occurring in Daptomycin- and Methicillin-Resistant Staphylococcus aureus Under Daptomycin Exposure
Source: Front Microbiol. 2020 Aug 14;11:1893. doi: 10.3389/fmicb.2020.01893 (PMC7456847; doi:10.3389/fmicb.2020.01893)
Supplement: Supplementary file 9 [file Data_Sheet_9.PDF]

Figure S4. Growth Curves

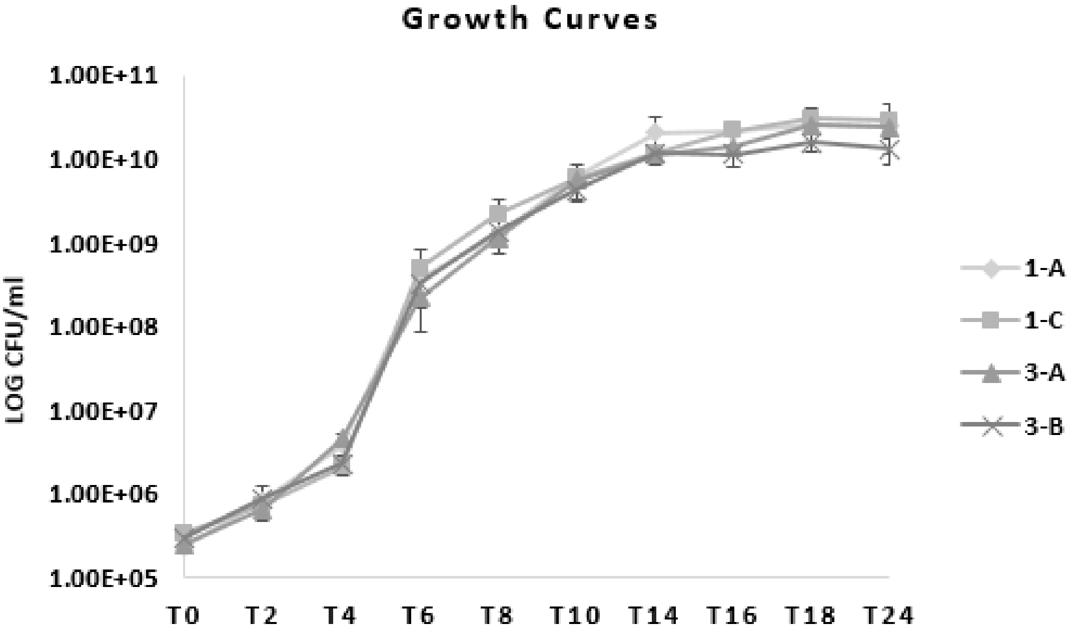

Showed values are the means of three replicates with standard deviation.

1A, 3A DAP^{S} MRSA

1C, 3B DAP^{R} MRSA
